# Combining bacterial display and protein language models to engineer a CD69-binding affibody for molecular imaging of immune activation

**DOI:** 10.1093/protein/gzag013

**Published:** 2026-06-11

**Authors:** Hugo Olsson, Cornelia Westerberg, Jonas Persson, Lina Löfstrand, Olle Korsgren, Olof Eriksson, Stefan Ståhl, John Löfblom

**Affiliations:** Department of Protein Science, Division of Protein Engineering, KTH Royal Institute of Technology, Roslagstullsbacken 21 SE-10691, Stockholm, Sweden; Department of Protein Science, Division of Protein Engineering, KTH Royal Institute of Technology, Roslagstullsbacken 21 SE-10691, Stockholm, Sweden; Department of Protein Science, Division of Protein Engineering, KTH Royal Institute of Technology, Roslagstullsbacken 21 SE-10691, Stockholm, Sweden; Department of Protein Science, Division of Protein Engineering, KTH Royal Institute of Technology, Roslagstullsbacken 21 SE-10691, Stockholm, Sweden; Department of Immunology, Genetics and Pathology, Uppsala University, Uppsala, SE-751 05, Sweden; Science for Life Laboratory, Department of Medicinal Chemistry, Uppsala University, Uppsala, SE-751 83, Sweden; Department of Protein Science, Division of Protein Engineering, KTH Royal Institute of Technology, Roslagstullsbacken 21 SE-10691, Stockholm, Sweden; Department of Protein Science, Division of Protein Engineering, KTH Royal Institute of Technology, Roslagstullsbacken 21 SE-10691, Stockholm, Sweden

**Keywords:** CD69, Affibody molecule, FACS, bacterial display, directed evolution, protein engineering, PET imaging, T cell activation

## Abstract

Recent years have seen remarkable clinical success with therapies that harness the patient’s immune system, with checkpoint inhibitors in oncology as a prominent example. Non-invasive monitoring of immune activation *in vivo* has the potential to accelerate both basic immunology research and clinical drug development, offering a valuable means to track responses to emerging immunotherapies. CD69 is a rapidly induced activation marker on lymphocytes and other leukocytes, making it an attractive imaging target. For such applications, affibody molecules offer distinct advantages as radio imaging tracers due to their small size, high affinity, and rapid pharmacokinetics, resulting in excellent imaging contrast. Here, we combined directed evolution with computational design to optimise a CD69-binding affibody molecule. An alanine scan of the parental binder informed the construction of a diversification library, which was displayed on *Escherichia coli* and subjected to iterative MACS and FACS selections with stringent off-rate competition. The top selection hit was subsequently refined through site-directed mutagenesis, including variants suggested by a general protein language model. The resulting lead, Z1525, bound human CD69 with single-digit nanomolar affinity and showed improved thermal stability while retaining solubility and refolding capacity, consistent with suitability for radiolabelling and *in vivo* targeting. Most importantly, it displayed selective binding to CD69 on stimulated Jurkat cells, with negligible binding to resting cells. These results establish *E. coli* display with off-rate–driven selection as an efficient strategy for affibody affinity maturation and demonstrate that protein language models can effectively guide improvements in folding stability. Z1525 represents a promising radio imaging tracer candidate for monitoring immune activation *in vivo* and warrants further preclinical development.

## 1. Introduction

Monitoring immune cell activation *in vivo* has the potential to provide valuable insight into immune system dynamics in health and disease. Development of non-invasive molecular imaging approaches with radiotracers, such as positron emission tomography (PET) or single-photon emission computed tomography (SPECT), could enable assessment of immune activation and dysregulation across a wide range of conditions (McCarthy *et al.*, 2020). Importantly, such tools could also allow direct evaluation of immune responses to therapeutic interventions. This need is becoming increasingly relevant as immunotherapy expands beyond checkpoint inhibitors in oncology, to include bispecific T cell engagers, NK cell engagers, and adoptive cell therapies such as CAR-T and CAR-NK cells (Fenis *et al.*, 2024; Peng *et al.*, 2024). These advanced therapeutic modalities depend on robust and well-timed immune activation, but current monitoring approaches remain largely invasive or indirect. Non-invasive tracers that can sensitively and specifically report on immune activation *in vivo* would therefore represent a valuable complement to existing diagnostics, supporting both basic immunology research and the clinical development of next-generation therapies.

Cluster of differentiation 69 (CD69) is an early activation marker of particular interest in immunology because of its rapid and transient expression on activated lymphocytes and its role in immune modulation (Cibrián and Sánchez-Madrid, 2017). On T cells, expression can be detected within two hours of stimulation and typically declines within 24 hours (Cibrián and Sánchez-Madrid, 2017; Radulovic and Niess, 2018). CD69 is constitutively expressed on cortical thymocytes, Langerhans cells, and platelets (Radulovic and Niess, 2018), and serves as a marker for tissue-resident memory T cells (Cibrián and Sánchez-Madrid, 2017). It is also upregulated on macrophages (Radulovic and Niess, 2018), eosinophils (Hartnell *et al.*, 1993; Radulovic and Niess, 2018), neutrophils (Atzeni *et al.*, 2002; Radulovic and Niess, 2018), NK cells (Lanier *et al.*, 1988), and B cells (Risso *et al.*, 1989) upon activation, whereas resting circulating leukocytes do not express the receptor (Radulovic and Niess, 2018). This combination of negligible baseline expression and rapid induction upon stimulation makes CD69 a suitable target for imaging of immune activation in contexts such as inflammation (Puuvuori *et al.*, 2024) and cancer immunotherapy (Edwards *et al.*, 2022; Nisnboym *et al.*, 2023). On the cell membrane, CD69 forms a disulphide-linked homodimer, consisting of two 28–32 kDa monomers, the exact weight depending on glycosylation (Testi *et al.*, 1994; Llera *et al.*, 2001).

Affibody molecules (Z domains) are small (~6–7 kDa), engineered affinity proteins with a three-helical fold that can be produced in *Escherichia coli* or by solid-phase peptide synthesis (Nord *et al.*, 1997; Ståhl *et al.*, 2017). In the context of *in vivo* molecular imaging, their small size promotes rapid tissue penetration and renal clearance, thereby reducing blood-pool activity and enhancing image contrast, enabling rapid tracer injection to imaging times, with image qualities comparing favourably to antibodies (Orlova *et al.*, 2009; Garousi *et al.*, 2016; Tolmachev and Orlova, 2020). Their simple and robust architecture also facilitates straightforward radiolabeling and reproducible manufacturing of tracers. Importantly, three affibody molecules have already advanced to clinical trials and been tested in more than 300 subjects, demonstrating excellent safety and activity profiles (www.affibody.se). Together, these properties provide a strong translational foundation for affibody-based radiotracers.

We have previously developed a CD69-specific affibody, which demonstrated promising characteristics as a radiotracer for imaging immune activation (Persson *et al.*, 2021; Puuvuori *et al.*, 2024). *In vivo* studies showed rapid clearance, low background, and focal uptake in lymphoid tissues consistent with CD69 engagement. In a longitudinal preclinical arthritis model, tracer uptake increased before overt clinical symptoms, correlating with disease severity, and aligned with *ex vivo* detection of CD69+ cells (Persson *et al.*, 2021; Puuvuori *et al.*, 2024). These findings suggest that CD69 imaging could non-invasively capture early tissue-level immune activation with a workflow compatible with clinical practice.

Building on these proof-of-concept data, the aim of the present study was to further improve CD69-specific affibodies to generate a candidate suitable for preclinical development. To this end, we employed our in-house *E. coli* display platform (Fleetwood *et al.*, 2014; Andersson *et al.*, 2019; Parks *et al.*, 2024) combined with fluorescence-activated cell sorting (FACS) for directed evolution of optimised CD69-binding variants. Selection conditions were adapted to include competition with the parental binder, thereby favouring variants with slower dissociation and higher affinity. This approach, together with computational design using a general protein language model, yielded a next-generation affibody with single-digit nanomolar affinity, favourable stability, and selective binding to CD69 expressed on activated cells in a Jurkat model, establishing a promising lead tracer for preclinical development and future clinical translation.

## 2. Materials and methods

### 2.1. *E. coli* display

BL21^*^  *E. coli* carrying the arabinose-inducible pPALU1 vector (Parks *et al.*, 2024), encoding affibody variants fused to an albumin-binding domain (ABD035) (Jonsson *et al.*, 2008), were cultivated in Luria–Bertani (LB) medium supplemented with 0.1 mg/mL carbenicillin. Overnight cultures were incubated at 37°C with shaking at 150 rpm. Cultures were diluted into fresh medium and grown to an optical density at 600 nm (OD600) of 0.5–0.8, after which expression was induced by addition of L-arabinose to a final concentration of 0.6% (w/v) for the alanine scan or 0.1% for selections and downstream analyses.

For the alanine scan, induced cultures were incubated overnight at 25°C with shaking at 150 rpm. For library selections, induction was performed for 1 h under the same conditions. Cells were harvested by centrifugation and resuspended in phosphate-buffered saline supplemented with 0.1% (w/v) pluronic acid (PBS-P) for subsequent analyses.

### 2.2. Design and assembly of the affinity maturation library

An alanine scan covering the 14 variable positions of the parental affibody ZCAM241 was performed using the *E. coli* display system described above. Following induction, cells were washed by centrifugation in PBS-P in a 96-well plate format and incubated for 45 min at room temperature with 50 nM biotinylated recombinant human CD69 (cat. no. 8468-CD, R&D Systems, Minneapolis, MN, USA) biotinylated using a Biotin-XX Microscale Protein Labeling Kit (cat. no. B30010, Invitrogen, Carlsbad, CA, USA) (hereafter referred to as hCD69-bio). After washing, cells were incubated for 30 min with 333 nM human serum albumin (HSA) conjugated to Alexa Fluor 647 and 2 μg/mL streptavidin–phycoerythrin (SAPE; cat. no. S866, Thermo Fisher Scientific, Waltham, MA, USA). After a final wash, 20 000 events per sample were acquired on a CytoFLEX S flow cytometer (Beckman Coulter, Brea, CA, USA) using 561/543–627 nm and 638/650–670 nm laser/filter combinations. An affinity maturation library was designed based on the alanine scan results and ordered from Ella Biotech (Fürstenfeldbruck, Germany) (Supplementary Table 1). The library incorporated three different mutation frequencies across the variable positions of ZCAM241, stratified according to the observed tolerance to alanine substitution. The library DNA was PCR-amplified, restriction-cloned into pPALU1 (Parks *et al.*, 2024), and electroporated across 20 aliquots into EXPRESS BL21 electrocompetent cells (cat. no. 60300–1, Biosearch Technologies, Hoddesdon, Great Britain). Following recovery in super optimal broth with catabolite repression (SOC) medium, electroporated cells were pooled and cultivated for 1 h at 37°C with shaking at 150 rpm, followed by overnight cultivation in tryptic soy broth (TSB) supplemented with 0.1 mg/mL carbenicillin. Library size was estimated by plating serial dilutions of the pooled electroporates on carbenicillin-supplemented LB agar plates and incubating overnight at 37°C. 48 colonies were sent for Sanger sequencing at Microsynth AG (Balgach, Switzerland) to verify the correct composition of the library.

### 2.3. MACS and FACS

Glycerol stocks containing at least a tenfold coverage of the affinity maturation library were used to inoculate *E. coli* display cultures as described above. Equivalent library coverage was maintained throughout all selection rounds. The library was first enriched using magnetic-activated cell sorting (MACS). Dynabeads MyOne Streptavidin C1 (cat. no. 65001, Thermo Fisher Scientific) were incubated with hCD69-bio for 1 h. Beads were washed twice in PBS-P using magnetic separation. Displaying *E. coli* were washed twice in PBS-P and subjected to a negative selection by incubation with non-labelled streptavidin-coated beads for 30 min. Cells remaining in the supernatant were washed and subsequently incubated with the hCD69-coated beads for 2 h. Beads were washed three times by magnetic separation and used to inoculate overnight cultures in LB supplemented with 0.1 mg/mL carbenicillin at 37°C and 150 rpm. Output library size was estimated by plating serial dilutions on agar plates.

Following MACS, four rounds of fluorescence-activated cell sorting (FACS) were performed. Displaying cells were incubated for 1 h with decreasing concentrations of hCD69-bio (50, 25, 10, and 5 nM) and at increasing temperatures (room temperature in round 1, 37°C in subsequent rounds). After target binding, cells were washed and subjected to off-rate incubation for increasing durations (starting from 1 h, increased to 6^*^30 min interspersed with washing and overnight incubation in FACS round 4), and with soluble ZCAM241 included as a competitor at increasing molar excess vis-à-vis hCD69-bio (100-fold in round 2, 500-fold in round 3, and either 0 or 500-fold in round 4). Cells were thereafter labelled on ice for 30 min with 333 nM HSA–Alexa Fluor 647 and either SAPE or NeutrAvidin–phycoerythrin (NAPE; both 2 μg/mL) and washed once more. Sorting was performed on a CytoFLEX SRT (Beckman Coulter), with laser/filter combinations of 561/543–627 nm and 638/650–670 nm, gating manually for the top binding fraction of cells. Sorted cells were recovered in LB medium for 1 h at 37°C, followed by overnight cultivation in LB with 0.1 mg/mL carbenicillin. Glycerol stocks were prepared for subsequent rounds.

After FACS rounds 3 and 4, individual clones were isolated (192 and 288 colonies, respectively), Sanger sequenced at Microsynth AG, and analysed by flow cytometry under conditions matching their respective selection rounds. At the conclusion of the campaign, all outputs were analysed by flow cytometry using the FACS round 4 protocol with ZCAM241 competition and four 30 min wash steps.

### 2.4. Production and purification of soluble affibodies

Affibodies to be analysed in solution (whether from selections or from subsequent mutation campaigns) were subcloned into the pET45b(+) production vector (Novagen/Merck, Darmstadt, Germany) containing an isopropyl β-D-1-thiogalactopyranoside (IPTG)-inducible T7 promoter, an Amp^R^ gene and a C-terminal hexahistidine tag using In-Fusion® Snap Assembly Master Mix (cat.no. 638949, Takara Bio, Kusatsu, Japan) according to the manufacturer’s instructions. Following heat shock transformation of BL21^*^, transformants were Sanger sequenced to confirm identity. Affibody-hexahistidine (Z-H_6_) expressing clones were cultivated overnight in TSB with 0.1 mg/mL carbenicillin, then diluted in TSB with yeast extract (TSB + Y) and 0.1 mg/mL carbenicillin. At 0.7 ≤ OD_600_ ≤ 1.0, cultures were induced with IPTG to a final concentration of 1 mM and incubated at 25°C and 150 rpm overnight before harvest and lysis through sonication. Lysates were purified through immobilized metal affinity chromatography (IMAC) at 4°C using HisPur Cobalt Resin (cat.no. 89966, ThermoFisher Scientific) and buffer-exchanged to PBS pH 7.4 on PD-10 columns (cat.no. 17085101, Cytiva, Uppsala, Sweden) in accordance with the manufacturer’s recommendations. Purified proteins were analysed for purity, concentration and identity (data not shown) by, respectively, sodium dodecylsulphate-polyacrylamide gel electrophoresis (SDS-PAGE) (NuPAGE, cat.no. NP0329BOX, Invitrogen), bicinchoninic acid assay (BCA) (cat.no. 23227, ThermoFisher Scientific) and mass spectrometry (MS), using matrix-assisted laser desorption ionization-time of flight (MALDI-TOF) (4800 MALDI-TOF/TOF, Applied Biosystems/ThermoFisher scientific).

### 2.5. Surface plasmon resonance for affinity screening and kinetics determination

Candidate affibodies were screened to identify the highest affinity binders on a Biacore 8 K (Cytiva) following selections and mutation campaigns. A Series S sensor chip CM5 (Cytiva, cat.no. 29149603) was immobilized with recombinant human CD69 (cat.no. 8468-CD, R&D Systems) dissolved in 10 mM pH 5.5 sodium acetate using Cytiva’s amine coupling kit (cat.no. BR100050) and aiming for 1000 response units (RU) employing the instrument’s immobilization wizard. Z-H_6_ samples ranging from 4–200 nM were injected over the chip surface for 200 s and allowed to dissociate for 600 s (180 s + 300 s for Z_A01_ double mutants) while the analysis temperature was maintained at 25°C. PBS with 0.05%–0.1% Tween20 (PBS-T) was used as running buffer and 100 mM glycine-HCl pH 2.1 was injected to regenerate the chip in between samples. A 1:1 Langmuir interaction model was fitted to the sensorgrams for affinity estimation, which together with a qualitative sensorgram comparison guided decisions on which candidates to proceed with. Representative Z-H_6_ constructs from each step of the affinity maturation were assessed kinetically on a Biacore T200 (Cytiva). Immobilization, buffer, temperature and regeneration conditions were as described above, immobilizing separate channels with recombinant human CD69-Fc and, as a negative control for the Fc tag, CLEC4C-Fc (cat.no. PME100606, PME100756, DIMA biotech, Suzhou, China) to levels of 1200 and 2000 RU, respectively. At concentrations ranging from 1–64 nM (or 15.625–1000 nM for ZCAM241) affibody molecules were injected for 500 s, followed by 350 s dissociation. The experiment was performed in triplicate and a 1:1 Langmuir curve fitting model was used to estimate kinetic parameters in the Biacore T200 software after manually trimming the curves of air spikes.

### 2.6. Biolayer interferometry for affinity screening

The Z-H_6_ constructs from the alanine scan of the affinity matured Z_A01_ were analysed in biolayer interferometry (BLI) on an Octet 96 Red (FortéBio, Fremont, CA, USA). Recombinant hCD69 (cat.no. 8468-CD, R&D Systems) dissolved in 10 mM acetate buffer pH 6.0 was coupled to ARG2-biosensors (cat.no. 18–5092, Sartorius, Göttingen, Germany) using NHS chemistry. Tips were submerged in 500 nM Z-H_6_ constructs dissolved in PBS-T for 900 s, followed by 900 s dissociation in PBS-T. 100 mM glycine-HCl pH 2.1 was used for regeneration between constructs. A blank sensor tip submerged in 500 nM Z_A01_, Z_A01-R13A_ or Z_A01-R18A_ (verified in pre-screening to be strong binders) was subtracted from remaining sensors and the signal of a hCD69-coated tip incubated in PBS-T was verified to be negligible.

### 2.7. Circular dichroism to analyse secondary structure and thermal stability

Z-H_6_ constructs were diluted to 0.1–0.3 mg/mL in PBS pH 7.4 and inserted into a Chirascan Circular Dichroism Spectrometer (Applied Biophysics, Troy, NY, USA), utilizing a 1 mm path length. The ellipticity from 195–260 nm was measured five times for secondary structure determination. This was followed by heat denaturation, raising the temperature from 20 to 95°C at 5°C/min, monitoring ellipticity at 221 nm. The thermal melting point (T_m_) was determined as the inflection point of a 4-parameter sigmoidal curve fit to the variable temperature measurement data using GraphPad Prism 10 (Dotmatics, Boston, MA, USA). Following cooling to below 25°C, the secondary structure determination was repeated to assess refolding.

### 2.8. Protein language algorithm and alanine scan employed to suggest single substitutions and used as basis for double mutants

The open-source code provided along with the publication *Efficient evolution of human antibodies from general protein language models* (Hie *et al.*, 2024) was cloned from GitHub to a local computer and run using Python 3 with the 59-character amino acid sequence of Z_A01_ as argument. The threshold likelihood ratio between suggested mutations and wild type amino acids in a given position, as defined by the language model, was set to 0.8. All mutations suggested by at least one of the six algorithms in the model’s ensemble were ordered as gene fragments from Twist Bioscience (South San Francisco, CA, USA), along with six additional mutants thought to encapsulate the chemical pattern of the suggested substitutions. In parallel, an alanine scan panel of the 15 variable (14 previous + insert) positions of Z_A01_ was also ordered from Twist Bioscience (Supplementary Table 5). Both campaigns followed the workflow outlined above for production, purification and CD spectroscopy, with the machine learning campaign undergoing affinity screening according to the SPR workflow and the alanine scan according to the BLI workflow, as described above. Substitutions from both tracks were combined into eight double mutants (Supplementary Table 6), which were ordered as gene fragments from Twist Bioscience and produced and analysed in the same way as the machine learning-derived single mutants.

### 2.9. Size exclusion chromatography

Affibody molecules ZCAM241, Z_A01_, Z_A01-W15E_, Z_A01-15E25A_ and Z_A01-K36A_ in Z-H_6_ format were analysed by size exclusion chromatography, using a Superdex 75 Increase 5/150 GL column (cat.no. 29148722, Cytiva) and a Gel Filtration Calibration Kit LMW (cat.no. 28403841, Cytiva) according to the manufacturer’s recommendations.

### 2.10. Activation of Jurkat cells to upregulate CD69 expression

Jurkat cells (ACC282, DSMZ, Braunschweig, Germany) were grown in 90% RPMI 1640 Medium (cat.no. 21875, Gibco, ThermoFisher Scientific) and 10% heat-inactivated fetal bovine serum (FBS) (cat.no. 17914671, Gibco, ThermoFisher Scientific) at 37°C, 5% CO_2_ and subcultured according to manufacturer’s recommendations. Twenty-four hours before flow cytometric analyses, approximately 2 × 10^6^ cells were diluted in 3 mL media in wells of a 6-well cell culture tissue culture plate (cat.no. 734–2323, VWR). 3 μL of eBioscience™ Cell Stimulation Cocktail (500×) (cat.no. 00–4970-93, ThermoFisher) was added to designated wells for cell stimulation, with matched wells with non-stimulated cells as control. The cells were then incubated at 37°C, 5% CO_2_ for 20–24 h.

### 2.11. Flow cytometry of Jurkat cells to study cell binding

For each sample, cells were collected and pooled from non-stimulated and cocktail-stimulated cell cultures, respectively, washed twice by centrifugation at 300 g, 4 min, and subsequently resuspended in 5 mL ice-cold PBS supplemented with 0.1% (w/v) bovine serum albumin (cat.no. A4503, Sigma-Aldrich/Merck) (PBS-B). After the second wash, the cells were diluted in PBS-B to a volume corresponding to ≤2 × 10^6^ cells/mL. For each sample, 200 μL of the respective cell suspension was added to the wells of a conical 96-well plate. The cells were pelleted by centrifugation (600 g, 2 min) and resuspended in 100 μL Z-H_6_ diluted in PBS-B to concentrations ranging from 1 μM to 125 nM. The labelling reaction proceeded for 45 min at room temperature under gentle rotation (150 rpm). The cells were washed and resuspended in 100 μL PBS-B supplemented with 2.5 μL CD69 Monoclonal Antibody (FN50), PE, eBioscience™ (cat.no. 12–0699-42, ThermoFisher Scientific) (positive control, no Z-H_6_ added) or His Tag Alexa Fluor® 488-conjugated Antibody (cat.no. MA1–135-A488, Invitrogen), diluted 1:500. The secondary labelling reaction proceeded for 30 min on ice, whereupon the cells were washed preceding flow cytometric analysis. A minimum of 20 000 events per sample were analysed by a CytoFLEX S (Beckman Coulter) with laser/filter combination 488/485–565 nm and 561/543–627 nm.

### 2.12. Next generation sequencing of sub-libraries

Glycerol stocks of the starting library and all selection outputs were cultivated overnight in LB with 0.1 mg/mL carbenicillin, and the cultures were subjected to miniprep with QIAprep® Spin Miniprep Kit (cat.no. 27106, QIAgen, Hilden, Germany). Following PCR with barcoded primers to amplify the affibody-encoding DNA, gel extraction ensued using the QIAquick® Gel Extraction Kit (cat.no. 28706, QIAgen). An agarose gel was run to verify the size and purity of the products and the DNA concentration was measured on a NanoDrop (model ND-1000, Thermo Scientific) (data not shown), whereafter they were pooled, with 2/3 of the total genetic material being derived from the starting library and equal parts of all sub-libraries making up the final third to account for the diversity of the starting library. The pooled DNA was sequenced using MiSeq (National Genomics Infrastructure, Stockholm, Sweden) and analysed with PipeBio (Horsens, Denmark). Sequences from the starting library and all selection cycles were annotated, filtered and subsequently clustered on 100% identity to reveal unique clones and their respective frequencies in the libraries. For the initial library, the per-position amino acid composition was computed. Clonal enrichment over the different selection cycles was analysed for the thirty most enriched variants.

## 3. Results

### 3.1. Alanine scan of a parental CD69 affibody molecule and design of maturation library

**Figure 1:**
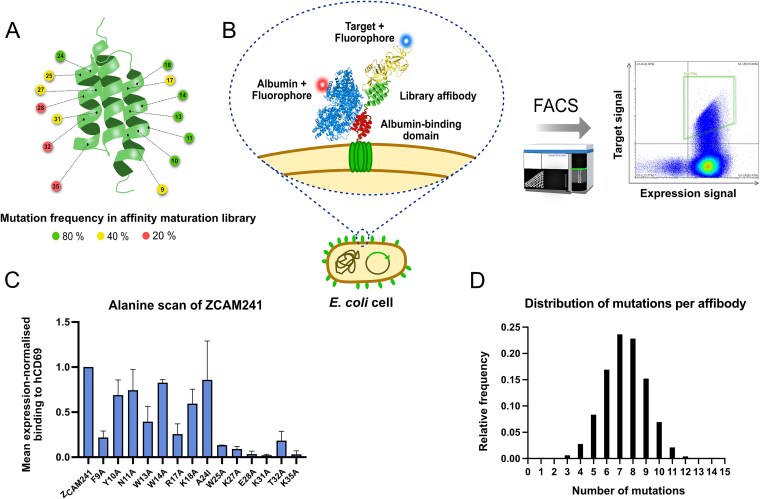
(A) Conceptual overview of the affibody molecule with the locations of randomized positions marked along with their assigned mutation frequencies in our affinity maturation library. (B) the principle of adhesin involved in diffuse adherence I (AIDA-I)-mediated *E. coli* display. An albumin binding domain (ABD_035_) (Jonsson *et al.*, 2008) is used for expression normalisation through binding to fluorescently labelled human serum albumin. N-terminally of this, the affibody molecule is displayed available for binding to target molecules fluorescently labelled through the biotin-streptavidin interaction. This dual fluorophore setup renders affibody clones sortable through FACS based on the quotient between the signals. Structures generated using AlphaFold 3 (Abramson *et al.*, 2024). (C) the result of an *E. coli* display-mediated alanine scan of Z_CAM241_. Results are the average of triplicate experiments. (D) the predicted frequency of different numbers of mutations per affibody resulting from our affinity maturation library design, calculated as the sum of three independent binomial distributions.

An alanine scan of the parental anti-CD69 affibody ZCAM241 (Persson *et al.*, 2021; Puuvuori *et al.*, 2024) was performed by systematically substituting each of its 14 randomized residues with alanine (Fig. 1A). The single-residue mutants were displayed individually on the surface of *E. coli* BL21^*^ using the autotransporter-based vector pPALU1 (Parks *et al.*, 2024), and their binding to fluorescently labelled recombinant human CD69 (hCD69) was assessed by flow cytometry. To account for variation in surface expression, binding signals were normalised to surface expression levels (quantified *via* binding of fluorescently labelled human serum albumin to the expression tag ABD035 (Jonsson *et al.*, 2008)), providing a relative measure of binding capacity for each variant (Fig. 1B).

These data guided the design of an affinity maturation library (Supplementary Table 1). Based on the alanine scan results (Fig. 1C), the 14 randomized positions of ZCAM241 were stratified into three categories with tailored mutation frequencies (Fig. 1A). Positions minimally affected by alanine substitution were assigned a parental residue frequency of 20%, with the remaining 80% distributed across a broad set of amino acids to maximize sequence diversity. Positions moderately affected by alanine substitution were assigned a 60% parental frequency, with the remaining 40% diversified among alternative amino acids. Positions least tolerant to substitution were assigned an 80% parental frequency, with non-parental residues restricted to conservative substitutions closely related to the parental identity. To further reduce the risk of excessive hydrophobicity, amino acid distribution at position N11 was adjusted to reduce hydrophobic residues, following principles described by Güler et al. (Güler *et al.*, 2020). Cysteine, glycine, and proline were excluded from all positions to avoid disulphide formation and helix destabilization, respectively. This strategy yielded a maturation library with an intended average of around 7 substitutions per affibody (Fig. 1D). Following PCR amplification and cloning into pPALU1, the library was transformed into *E. coli* BL21^*^, yielding a displayed library size of ~1 × 10^9^ variants as estimated from colony counts of electroporated cells (Supplementary Table 2). Per-residue mutation frequencies were initially assessed by Sanger sequencing (Supplementary Table 3) and were consistent with the intended design, which was later confirmed by MiSeq analysis (Supplementary Fig. 1).

### 3.2. MACS and FACS of the *E. coli*-displayed affinity maturation library

**Figure 2:**
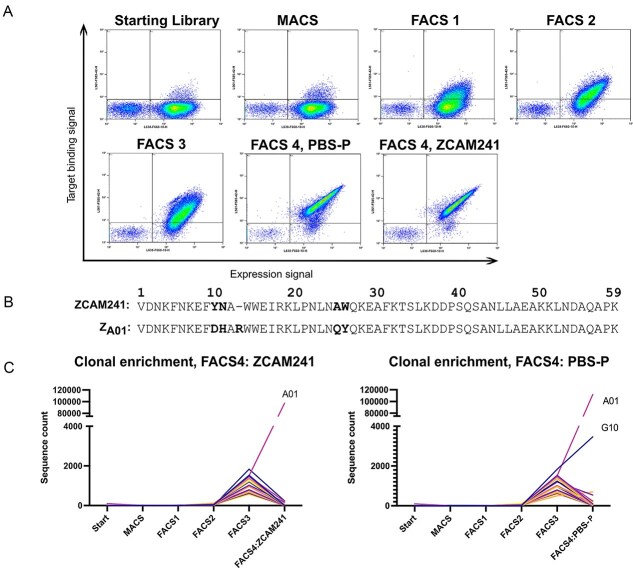
(A) Dot plots analysing the *E. coli* display sub-libraries emerging from subsequent sorting rounds of the maturation library. The x-axis shows fluorescence intensity for the 638/650–670 nm laser/filter combination, corresponding to the degree of surface display and the y-axis shows fluorescence intensity for the 561/543–627 nm combination, proportional to the degree of hCD69 binding to displaying *E. coli*. The upwards moving trend of the bacterial populations throughout the selection campaign indicates an enrichment in hCD69 binding. Wash buffers for the two FACS round 4 tracks are stated in sub-figure titles. (B) Amino acid sequence alignment between the starting sequence ZCAM241 and the most enriched binder in FACS 4, Z_A01_, showing the differences in bold and a gap in ZCAM241 to denote the unintended insertion found in Z_A01_. (C) Enrichment of the individual sequences through the selection rounds, as determined by MiSeq. The left pane shows the enrichment pattern for the arm that was washed with an excess of ZCAM241 in FACS round 4; the right pane shows the same for the arm washed with PBS-P in FACS round 4.

The displayed library was subjected to selections against biotinylated recombinant human CD69 (hCD69-bio hereafter), beginning with magnetic-activated cell sorting (MACS). A single MACS round reduced the library to approximately 7 × 10^5^ cells, with a modest enrichment of the hCD69-binding fraction (<2-fold), as assessed by flow cytometry using fluorescently labelled hCD69 (Fig. 2A). Given the high multivalency of *E. coli* display and the dimeric nature of hCD69, avidity effects were expected to compromise the ability to impose effective selection pressure for slow dissociation rates during FACS.

To address this, a strategy was evaluated in which excess soluble parental binder, ZCAM241 in Z-hexahistidine (Z-H_6_) format, was added in the washing step after incubating cells with hCD69-bio. The rationale was that hCD69 molecules dissociating from displayed binders would be captured by soluble ZCAM241, thereby reducing re-binding and potentially enriching for variants with slow dissociation. The feasibility of this approach was first confirmed using *E. coli* displaying ZCAM241, where addition of soluble ZCAM241 produced a dose-dependent reduction in the fluorescence signal corresponding to labelled hCD69 binding (Supplementary Fig. 2A and B). Comparison with the addition of non-labelled (‘cold’) hCD69, a standard method to prevent re-binding, indicated that the soluble parental binder imposed a more stringent and cost-effective selection pressure (Supplementary Fig. 2C).

The MACS-enriched library was subsequently subjected to FACS. Progressive enrichment of hCD69-binding clones was observed over successive rounds, as monitored by flow cytometry (Fig. 2A). In FACS round 4, two parallel selection tracks were performed, differing in the off-rate incubation buffer: track 1 used PBS-P, while track 2 included a 500-fold molar excess of soluble ZCAM241 over hCD69-bio, according to the strategy described above.

More than 400 clones from FACS rounds 3 and 4 were analysed by Sanger sequencing. Round 3 sequencing identified 141 unique sequences, with no single sequence occurring more than three times, indicating high diversity (Supplementary Fig. 3). In contrast, sequencing of 192 clones from track 1 of round 4 and 96 clones from track 2 revealed a dominant clone, designated Z_A01_, which accounted for 91% and 100% of reads, respectively. Track 1 also yielded eight additional unique sequences at low frequencies (one to five occurrences). Notably, Z_A01_ carried an unintended amino acid insertion not part of the maturation library design (Supplementary Table 4; Fig. 2B), as did Z_G10_, another clone isolated in FACS round 4. Next-generation sequencing (NGS) of all sub-libraries corroborated the Sanger sequencing results, confirming the strong enrichment of Z_A01_ in round 4 and the absence of comparable enrichment after round 3 (Fig. 2C).

### 3.3. SPR and CD characterisation of affibody variants from FACS round 4

**Figure 3:**
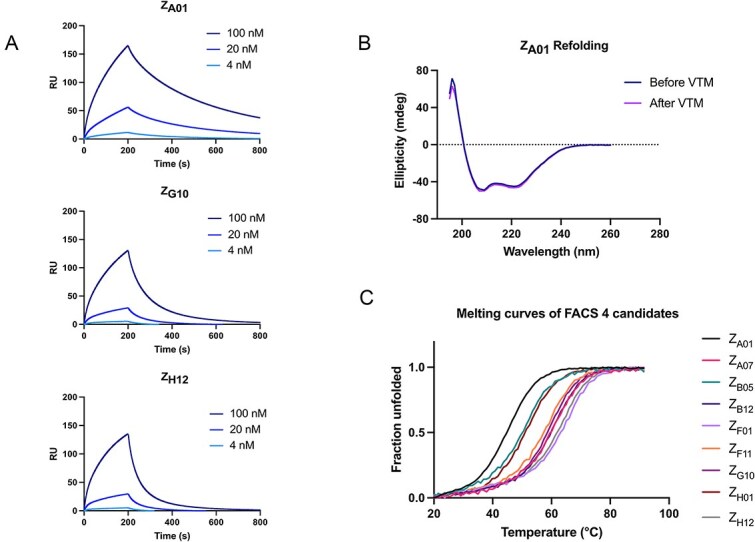
Characterisation of individual clones after FACS 4. Z_A01_ stood out as the most promising candidate from an affinity perspective, but its low T_m_ motivated further development. (A) Sensorgrams from SPR injecting affibodies as Z-H_6_ over hCD69 for three of the clones displaying slow dissociation, including Z_A01_. (B) Overlapped CD spectra of Z_A01_ before and after heat denaturation. Z_A01_ displays the characteristic refolding behaviour common to affibody molecules. (C) CD melting curves of the nine individual affibody clones sequenced after FACS 4.

The nine clones identified after FACS round 4 were subcloned into the Z-H_6_ format, produced in *E. coli* BL21^*^, and purified for characterisation. Binding kinetics were evaluated by surface plasmon resonance (SPR) using immobilized hCD69 and analyte injections of 4, 20, and 100 nM affibody variants. Z_A01_ displayed markedly slower dissociation compared with the other candidates (Fig. 3A; Supplementary Fig. 4). Secondary structure content, thermostability, and refolding capacity were assessed by circular dichroism (CD) spectroscopy. All variants exhibited the expected alpha-helical spectra and demonstrated reversible refolding after heat denaturation (Fig. 3B; Supplementary Fig. 5A). Variable temperature CD spectroscopy revealed melting temperatures (T_m_) ranging from 44 to 60°C, with Z_A01_ displaying the lowest T_m_ of 44°C (Fig. 3C; Supplementary Fig. 5B). Despite its comparatively low thermal stability, the strong clonal enrichment combined with slow dissociation kinetics identified Z_A01_ as the primary candidate for further development.

### 3.4. Design of Z_A01_ single-residue substitution variants

To improve the stability of Z_A01_ while retaining affinity, we explored two complementary strategies for single-residue substitutions. First, we evaluated a protein language model recently developed by Kim and co-workers (Hie *et al.*, 2024). Although originally presented as an antibody engineering tool primarily aimed at affinity maturation, the developers also reported examples of stabilisation. The model predicts single amino acid substitutions based on evolutionary patterns learned from large datasets of natural protein sequences, without being explicitly trained to optimise any particular biophysical property. We therefore hypothesised that the model could also identify substitutions beneficial for affibody stability and/or affinity. Application of the model to the Z_A01_ sequence yielded six suggested single substitutions: W15A, W15D, W15E, W15N, Y26A, and Y26D. Noting a preference for substitutions of W15 to non-hydrophobic residues, we expanded this set by including five additional variants at this position (W15R, W15K, W15S, W15T, and W15Q). No further substitutions were introduced at position Y26, since this position had shown limited variability in the FACS round 3 output, whereas substantial variation was observed at position 15 (Supplementary Fig. 3; note that positions 15 and 26 in Z_A01_ correspond to 14 and 25 in the parental sequence due to the insertion in Z_A01_).

In parallel, an alanine scan of Z_A01_ was conducted, in which each of the 14 positions randomized in the maturation library, as well as the insertion residue R13, was individually replaced by alanine, yielding 15 additional variants. Together with the protein language model–derived variants, and accounting for overlap between the two sets, a total of 24 single-residue substitution variants of Z_A01_ were selected (Supplementary Table 5). All variants were cloned into the Z-H_6_ format, produced in *E. coli* BL21^*^, purified, and subjected to affinity and stability characterisation.

### 3.5. Characterisation of Z_A01_ single-residue substitution variants

The binding kinetics of the protein language model–derived single mutants were evaluated by SPR. Variants with substitutions at W15 generally displayed kinetic profiles comparable to Z_A01_, whereas substitutions at Y26 caused substantial reductions in affinity (Supplementary Fig. 6). All variants demonstrated reversible refolding after thermal denaturation in CD spectroscopy (Supplementary Fig. 7A) but exhibited variable thermostability (Supplementary Figs. 7B and 13A). Among the language model–derived variants, Z_A01-W15A_, Z_A01-W15D_, Z_A01-W15E_, and Z_A01-W15N_ combined preserved binding profiles with increased thermal stability. Z_A01-W15E_ was of particular interest, displaying a 9°C increase in melting temperature compared with Z_A01_ (53°C *vs.* 44°C) while maintaining target affinity. Additional W15 variants retained binding but showed modest to no improvements in thermostability (43–47°C).

The kinetic profiles of the 15 alanine-substitution variants of Z_A01_ were analysed by biolayer interferometry (BLI). Two substitutions (K32A and K36A) completely disrupted binding to hCD69. Five substitutions (R13A, W15A, R18A, K19A, and Q25A) rendered kinetic profiles similar to Z_A01_ (Supplementary Fig. 8). Thermostability and refolding capacity were assessed by CD spectroscopy, confirming reversible refolding for all variants except E29A (Supplementary Fig. 9A). Substitutions R13A and R18A markedly increased thermostability, with melting temperatures of 57°C and 50°C, respectively, whereas other substitutions, such as K28A, substantially decreased stability (Supplementary Fig. 9B, Supplementary Fig. 13B).

### 3.6. Design and characterisation of Z_A01_ double mutants

**Figure 4:**
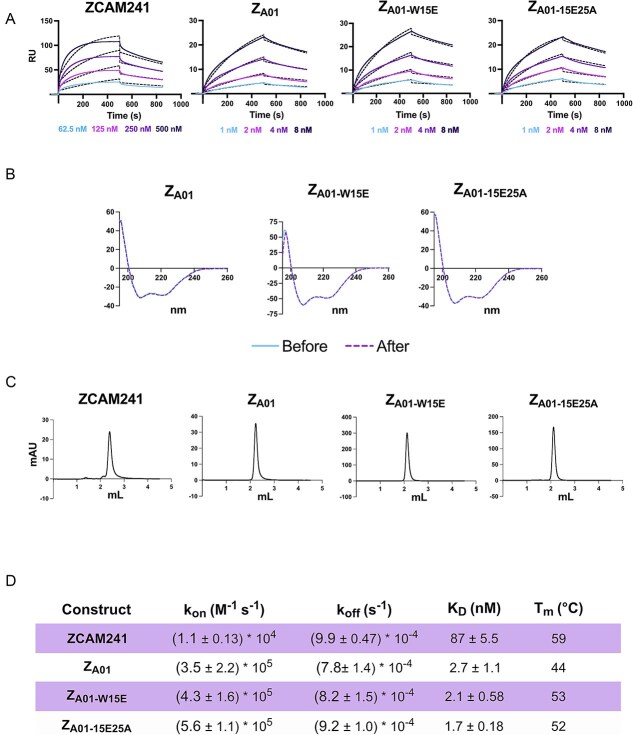
Characterisation of top candidates from the four different steps of the affinity maturation: Starting point (ZCAM241), selection output (Z_A01_), Z_A01_ single mutants (Z_A01-W15E_) and Z_A01_ double mutants (Z_A01-15E25A_). (A) SPR sensorgrams as full lines and 1:1 fits to determine kinetic parameters as dotted lines. The ligand used was hCD69-fc. (B) Complete refolding was observed for all three Z_A01_-based constructs in CD spectroscopy. (C) All binders appeared dominantly monomeric in SEC, eluting together with their expected size standard (Supplementary Fig. 12D). (D) Overview of kinetic parameters and thermal stability for the four assayed binders.

Next, eight double mutants combining beneficial substitutions from the single mutant screen were analysed (Supplementary Table 6): Z_A01-13A15E_, Z_A01-13A15N_, Z_A01-13A18A_, Z_A01-13A19A_, Z_A01-13A25A_, Z_A01-15E18A_, Z_A01-15E19A_, and Z_A01-15E25A_. These variants were produced, purified, and characterised for hCD69 affinity, refolding, and thermostability as for the previous mutant panels (Supplementary Figs. 10, 11, and  13C). The most thermostable variant, Z_A01-13A18A_, reached a T_m_ of 65°C compared with 44°C for Z_A01_, but displayed reduced affinity for hCD69. This reduction in affinity was generally observed across the double mutants, with the exception of Z_A01-15E25A_, which showed binding comparable to Z_A01_ in the SPR screen. With a T_m_ of 52°C, this variant was selected as the top double mutant candidate and subjected to detailed analysis together with ZCAM241, Z_A01_, Z_A01-W15E_, and a negative control (Z_A01-K36A_) (Fig. 4; Supplementary Fig. 12).

In SPR, Z_A01_, Z_A01-W15E_, and Z_A01-15E25A_ all displayed similar affinities for hCD69, with equilibrium dissociation constants (K_D_) of ~2 nM, representing an approximately 50-fold improvement compared with ZCAM241 (Fig. 4A). None of the constructs showed measurable binding to the Fc control flow cell, except for minor interaction at high concentrations for ZCAM241 (Supplementary Fig. 12A). All Z_A01_-based variants demonstrated complete refolding in CD spectroscopy (Fig. 4B) and monomeric behaviour in size exclusion chromatography (SEC) (Fig. 4C). Based on its combined properties of high CD69 affinity, thermostability, and refolding capacity, Z_A01-15E25A_ (Z1525 from here on) was selected as the final candidate (Fig. 4D).

### 3.7. Binding of candidates to CD69 on activated Jurkat T cells

**Figure 5:**
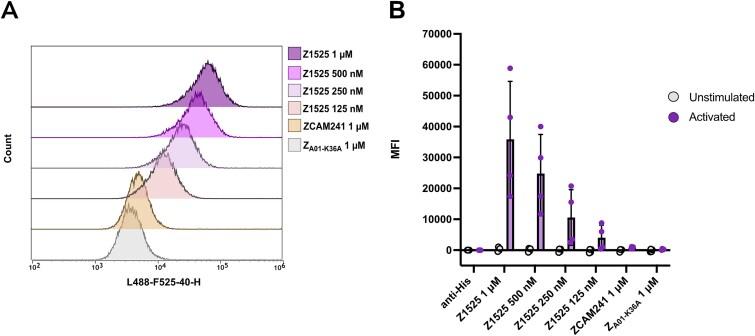
Flow cytometric analysis of unstimulated and activated Jurkat cells incubated with different concentrations (125 nM - 1 μM) of candidate Z1525 or the corresponding highest concentration of precursor ZCAM241 or null variant Z_A01-K36A_. (A) Overlay histogram of the different samples for one representative flow cytometric analysis assay. (B) Bar chart of the median fluorescent intensities (MFI) normalised against the MFI of the anti-his A488 control.

To evaluate the ability of Z1525 to bind CD69 on human cells, we used the immortalized leukemic T cell line Jurkat as a model. As Jurkat cells express CD69 only at low basal levels, cells were stimulated with phorbol myristate acetate and ionomycin to induce CD69 expression, and flow cytometric analyses were performed on resting and activated cells stained with either the precursor ZCAM241 or the top candidate Z1525. An anti-CD69 antibody served as a positive control for induction of CD69 expression (Supplementary Fig. 14). Z1525 bound in a titratable manner to activated, but not to resting, Jurkat cells (Fig. 5). This selectivity is consistent with the intended targeting profile, as it demonstrates that the affibody engages CD69, which is only expressed on activated cells. In line with the kinetic studies, Z1525 displayed stronger binding to activated cells than ZCAM241. As a negative control, we included Z_A01-K36A,_ which had shown no binding to recombinant hCD69 in SPR, and no binding to Jurkat cells was indeed observed. Taken together, Z1525 showed clearly improved and specific binding to hCD69 on activated human T cells compared with ZCAM241.

## 4. Discussion

Non-invasive imaging of immune activation could advance immunology research and clinical drug development by enabling dynamic assessment of immune responses *in vivo* (McCarthy *et al.*, 2020). We have previously reported promising *in vivo* results with a CD69-specific affibody, ZCAM241, demonstrating its potential as a tracer for such purposes (Persson *et al.*, 2021; Puuvuori *et al.*, 2024). Building on this proof-of-concept, the present study aimed to generate next-generation CD69-binding affibodies with the biophysical and functional properties required for translational development.

Through a combination of *E. coli* display–based directed evolution and computational design, we identified Z1525, a variant with excellent affinity and increased stability. Importantly, it bound hCD69 on activated T cells but not on resting ones, showing that the affinity-matured binder has the biological specificity required for future imaging of immune activation *in vivo*.

The enrichment of Z_A01_ highlights both the opportunities and challenges of using *E. coli* display for selections against multimeric targets. Cell display systems offer important advantages compared with traditional biopanning, as they allow for fluorescence-based monitoring and sorting of large libraries by flow cytometry. This enables quantitative tracking of enrichment, fine control over sorting parameters, and ranking of hits directly on the cytometer, prior to subcloning for characterisation of soluble proteins. *E. coli* in particular provides an attractive cell surface display platform because of its high transformation efficiency, and we have previously shown that the AIDA-I autotransporter supports robust display of affibody molecules (Fleetwood *et al.*, 2014; Andersson *et al.*, 2019; Parks *et al.*, 2024). At the same time, avidity effects inherent to multivalent surface display on cells can obscure differences in dissociation kinetics, particularly for dimeric receptors such as CD69 (Testi *et al.*, 1994; Llera *et al.*, 2001), where high affibody density permits rebinding and thereby interferes with the selection pressure needed to enrich for slow-dissociating, high-affinity binders. In unpublished work, we found that shortening the induction protocol lowered expression density, but in this study, this adjustment alone was insufficient to discriminate variants based on off-rates. Only when a stringent washing protocol was introduced in the final FACS round, including competition with soluble parental binder ZCAM241, did Z_A01_ emerge as the dominant clone in the output. Notably, this strategy was more effective than adding excess unlabelled hCD69 during washes. The difference likely lies in the mode of competition; soluble ZCAM241 captures hCD69 in solution and thereby prevents it from rebinding to the bacterial surface, whereas cold hCD69 binds free surface-expressed affibodies, reducing the number of available binding sites but seemingly with less influence on rebinding events. We deliberately designed the selection strategy to favour variants with slow dissociation kinetics, reasoning that prolonged target retention would be advantageous for molecular imaging applications and that rebinding effects inherent to multivalent display complicate selection for low off-rates. Such avidity- and rebinding-related effects are a general challenge in cell display systems, including yeast display, highlighting the value of developing and comparing strategies that reduce target rebinding during selections. At the same time, alternative selection strategies could potentially favour variants with different kinetic profiles, and future studies comparing selection schemes emphasising slow dissociation *versus* rapid association kinetics could therefore be of interest. The dominant Z_A01_ was indeed distinguished from the other selected variants by its slower dissociation, demonstrating that the selection pressure was effectively transferred into the desired kinetic property. This correspondence between applied pressure and output characteristics illustrates how display selections can be adjusted to overcome the limitations of multivalent presentation. In addition to selections, we also employed *E. coli* display for the alanine scan that informed the maturation library design, further illustrating the versatility of this platform for protein engineering.

An unanticipated outcome of the maturation process was the emergence of an unintended insertion in helix 1 of Z_A01_. Notably, the second-best clone identified (Z_G10_) also harboured a helix 1 insertion, suggesting that some degree of local helix reorientation may favour the interaction with hCD69. While such indels are not typically included in rational library designs, this outcome highlights the capacity of display-based directed evolution to enrich beneficial sequence alterations that lie outside the library design. This also illustrates the strength of directed evolution approaches, where a certain element of serendipity can reveal productive solutions that might be overlooked by purely rational or computational strategies. At the same time, the observed mutational load of the enriched clones suggests that our library design could be further optimised. Whereas the non-selected library was designed to have an expect value of seven substitutions per affibody, the functional binders that emerged carried fewer, with none exceeding six mutations, indicating a preference for lower mutational loads. More conservative mutation frequencies might have increased the proportion of functional variants, thereby improving overall efficiency.

Our workflow also illustrates the complementarity of directed evolution and computational design. Computational design using a protein language model (Hie *et al.*, 2024), together with experimental alanine scanning, subsequently enabled refinement of Z_A01_, the most enriched binder from selections, into Z1525, combining excellent affinity with increased stability. To further leverage the predictive power of the model, we complemented the protein language model suggestions at position W15 with an additional control set of substitutions. These were not strictly rational designs but rather a systematic extension of the mutational space favoured by the algorithm. Whereas the language model substitutions consistently stabilized the scaffold (T_m_ 48–53°C, with W15E increasing stability by 9°C), the control set produced neutral or destabilizing effects (T_m_ 43–47°C). These results suggest that the protein language model was able to identify substitutions beneficial for affibody stability, despite not being explicitly trained or designed for thermostability engineering. Importantly, the final mutational design was also guided by prior knowledge of sequence tolerability derived from display selections when choosing which suggested positions from the language model to diversify further. Position 26 was suggested for diversification by the model, but sequencing data of 192 clones from FACS round 3 indicated that only hydrophobic residues were tolerated at this site (Supplementary Fig. 3), leading us to avoid evaluation of any Y26 mutants beyond the algorithm’s direct suggestions. Indeed, these mutations (Y26A and Y26E) practically abrogated binding to hCD69 (Supplementary Fig. 6). Thus, positions W15 and Y26 were explored to different extents, reflecting a focused experimental strategy guided by both computational predictions and empirical sequence tolerance data. More broadly, sequencing after FACS round 3 corroborated the results of the primary alanine scan, with the same positions that tolerated alanine substitutions retaining diversity and *vice versa* (Fig. 1C, Supplementary Fig. 3). This empirical knowledge of the affibody paratope served as useful constraints to our focused investigation of the computational predictions.

Moreover, this study also underlines how assay choice influences the interpretation of apparent improvements. ZCAM241, despite optimisation of our biosensor measurements, dissociates in a biphasic manner with a rapid initial drop followed by a slower phase. Because the fitting procedure for K_D_ estimation is weighted toward this second phase (Fig. 4A), the kinetic constants underestimate the difference between ZCAM241 and Z1525. In contrast, cell binding assays provided a more biologically relevant measure of performance. Here, ZCAM241 resembled the negative control, whereas Z1525 exhibited around 10 000-fold higher median fluorescence intensity at equimolar concentrations.

To further evaluate Z1525 as a molecular imaging tracer, relevant next steps would include radiolabelling, biodistribution studies, and PET imaging of immune activation *in vivo*. Such studies will determine how the favourable *in vitro* properties of Z1525, including high affinity, selective binding to activated cells, and thermal stability compatible with radiolabelling, translate to *in vivo* imaging performance. The combination of CD69’s rapid induction kinetics and the favourable pharmacokinetics of affibodies supports the potential of this binder as a PET tracer (Orlova *et al.*, 2009; Garousi *et al.*, 2016; Cibrián and Sánchez-Madrid, 2017; Tolmachev and Orlova, 2020). Particularly in the context of cancer immunotherapy, where early assessment of immune activation is closely linked to treatment response and survival (Nisnboym *et al.*, 2023), Z1525 could provide a valuable tool for non-invasive monitoring of immune responses.

## Supplementary Material

Supplementary_Data_gzag013

## Data Availability

The data underlying this article are available in the article and in its online supplementary material.
