# Supplementary material for: Combining bacterial display and protein language models to engineer a CD69-binding affibody for molecular imaging of immune activation
Source: Protein Eng Des Sel. 2026 Jun 11;39:gzag013. doi: 10.1093/protein/gzag013 (PMC13284772; doi:10.1093/protein/gzag013)
Supplement: Supplementary_Data_gzag013 [file supplementary_data_gzag013.pdf]

Supplementary Data

Combining bacterial display and protein language models to engineer a CD69-binding affibody for molecular imaging of immune activation

Hugo Olsson[1*], Cornelia Westerberg[1*], Jonas Persson[1], Lina Löfstrand[1], Olle Korsgren[3], Olof Eriksson[2], Stefan Ståhl[1], John Löfblom[1#]

* Contributed equally

# Corresponding author: lofblom@kth.se

Supplementary Table 1: Per position composition of the affinity maturation library design.

**Amino acid in ZCAM241**	**Position**	**Amino acid composition in maturation library**
F	9	F(60%), A(2.5%), D(2.5%), E(2.5%), H(2.5%), I(2.5%), K(2.5%), L(2.5%), M(2.5%), N(2.5%), Q(2.5%), R(2.5%), S(2.5%), T(2.5%), V(2.5%), W(2.5%), Y(2.5%)
Y	10	Y(20%), A(5%), D(5%), E(5%), F(5%), H(5%), I(5%), K(5%), L(5%), M(5%), N(5%), Q(5%), R(5%), S(5%), T(5%), V(5%), W(5%)
N	11	N(20%), D(8.89%), E(8.89%), H(8.89%), K(8.89%), Q(8.89%), R(8.89%), S(8.89%), T(8.89%), Y(8.89%)
W	13	W(20%), A(5%), D(5%), E(5%), F(5%), H(5%), I(5%), K(5%), L(5%), M(5%), N(5%), Q(5%), R(5%), S(5%), T(5%), V(5%), Y(5%)
W	14	W(20%), A(5%), D(5%), E(5%), F(5%), H(5%), I(5%), K(5%), L(5%), M(5%), N(5%), Q(5%), R(5%), S(5%), T(5%), V(5%), Y(5%)
R	17	R(60%), A(2.5%), D(2.5%), E(2.5%), F(2.5%), H(2.5%), I(2.5%), K(2.5%), L(2.5%), M(2.5%), N(2.5%), Q(2.5%), S(2.5%), T(2.5%), V(2.5%), W(2.5%), Y(2.5%)
K	18	K(20%), A(5%), D(5%), E(5%), F(5%), H(5%), I(5%), L(5%), M(5%), N(5%), Q(5%), R(5%), S(5%), T(5%), V(5%), W(5%), Y(5%)

A	24	A(20%), D(5%), E(5%), F(5%), H(5%), I(5%), K(5%), L(5%), M(5%), N(5%), Q(5%), R(5%), S(5%), T(5%), V(5%), W(5%), Y(5%)
W	25	W(60%), A(2.5%), D(2.5%), E(2.5%), F(2.5%), H(2.5%), I(2.5%), K(2.5%), L(2.5%), M(2.5%), N(2.5%), Q(2.5%), R(2.5%), S(2.5%), T(2.5%), V(2.5%), Y(2.5%)
K	27	K(60%), A(2.5%), D(2.5%), E(2.5%), F(2.5%), H(2.5%), I(2.5%), L(2.5%), M(2.5%), N(2.5%), Q(2.5%), R(2.5%), S(2.5%), T(2.5%), V(2.5%), W(2.5%), Y(2.5%)
E	28	E(80%), D(20%)
K	31	K(80%), R(80%)
T	32	T(60%), A(2.5%), D(2.5%), E(2.5%), F(2.5%), H(2.5%), I(2.5%), K(2.5%), L(2.5%), M(2.5%), N(2.5%), Q(2.5%), R(2.5%), S(2.5%), V(2.5%), W(2.5%),Y(2.5%)
K	35	K(80%), R(80%)

Supplementary Table 2: Affinity maturation library size estimated from counting the number of colonies on agar plates streaked with dilutions of electroporated BL21* *E. coli.* The average of the 10^4 and 10^5 estimates was taken as the library size.

Dilution factor	I	II	Mean	Cells/mL	Library size estimation
10^4	142	180	161	16 100 000	8.05E8
10^5	19	31	25	25 000 000	1.25E9
10^6	0	3	1.5	15 000 000	7.5E8
10^7	1	0	0.5	50 000 000	2.5E9

Supplementary Table 3: Per position mutation frequency of the variable positions in the transformed library, as estimated by Sanger sequencing of 48 clones. The denominators differ between positions since the number of reads excluded due to ambiguity varied between positions.

Amino acid in Z_{CAM241}	Position	Mutation frequency (quotient)	Intended mutation frequency (%)
F	9	31% (10/32)	40
Y	10	69% (22/32)	80
N	11	74% (26/35)	80
W	13	71% (22/31)	80
W	14	81% (25/31)	80
R	17	44% (14/32)	40
K	18	74% (25/34)	80
A	24	65% (20/31)	80
W	25	38% (12/32)	40
K	27	31% (10/32)	40
E	28	27% (10/37)	20
K	31	18% (7/40)	20
T	32	39% (12/31)	40
K	35	23% (9/39)	20

Supplementary Table 4: Sequences of individual affibody clones identified after FACS 4.

Name	Sequence
ZA01	VDNKFNKEFDHARWWEIRKLPNLNQYQKEAFKTSLKDDPSQSANLLAEAKKLNDAQAPK
ZA07	VDNKFNKEFHYAWHEIRRLPNLNAWQKEAFKTSLKDDPSQSANLLAEAKKLNDAQAPK
ZB05	VDNKFNKEIHNAWIEIRILPNLNIWQKEAFKTSLKDDPSQSANLLAEAKKLNDAQAPK
ZB12	VDNKFNKEFHQAWSEIRKLPNLNIWQKEAFKTSLKDDPSQSANLLAEAKKLNDAQAPK
ZF01	VDNKFNKEFFDAWAEIRKLPNLNAWQKEAFKTSLKDDPSQSANLLAEAKKLNDAQAPK
ZF11	VDNKFNKEFLDAWSEIRHLPNLNIWQKEAFKTSLKDDPSQSANLLAEAKKLNDAQAPK
ZG10	VDNKFNKEFNHSAWAEIRALPNLNAWQKEAFKTSLKDDPSQSANLLAEAKKLNDAQAPK
ZH01	VDNKFNKEMAYAWTEIRHLPNLNIWQKEAFKTSLKDDPSQSANLLAEAKKLNDAQAPK
ZH12	VDNKFNKEMLSAWAEIKKLPNLNQWQKEAFKTSLKDDPSQSANLLAEAKKLNDAQAPK

Supplementary Table 5: Sequences of the single mutants of Z_{A01} evaluated.

Name	Sequence
ZA01-W15A	VDNKFNKEFDHARWAEIRKLPNLNQYQKEAFKTSLKDDPSQSA NLLAEAKKLNDAQAPK
ZA01-W15D	VDNKFNKEFDHARWDEIRKLPNLNQYQKEAFKTSLKDDPSQSA NLLAEAKKLNDAQAPK
ZA01-W15E	VDNKFNKEFDHARWEEIRKLPNLNQYQKEAFKTSLKDDPSQSAN LLAEAKKLNDAQAPK
ZA01-W15K	VDNKFNKEFDHARWKEIRKLPNLNQYQKEAFKTSLKDDPSQSA NLLAEAKKLNDAQAPK
ZA01-W15N	VDNKFNKEFDHARWNEIRKLPNLNQYQKEAFKTSLKDDPSQSA NLLAEAKKLNDAQAPK
ZA01-W15Q	VDNKFNKEFDHARWQEIRKLPNLNQYQKEAFKTSLKDDPSQSA NLLAEAKKLNDAQAPK
ZA01-W15R	VDNKFNKEFDHARWREIRKLPNLNQYQKEAFKTSLKDDPSQSA NLLAEAKKLNDAQAPK
ZA01-W15S	VDNKFNKEFDHARWSEIRKLPNLNQYQKEAFKTSLKDDPSQSAN LLAEAKKLNDAQAPK
ZA01-W15T	VDNKFNKEFDHARWTEIRKLPNLNQYQKEAFKTSLKDDPSQSAN LLAEAKKLNDAQAPK
ZA01-Y26A	VDNKFNKEFDHARWWEIRKLPNLNQAQKEAFKTSLKDDPSQSA NLLAEAKKLNDAQAPK
ZA01-Y26E	VDNKFNKEFDHARWWEIRKLPNLNQEQKEAFKTSLKDDPSQSA NLLAEAKKLNDAQAPK
ZA01-F9A	VDNKFNKEADHARWWEIRKLPNLNQYQKEAFKTSLKDDPSQSA NLLAEAKKLNDAQAPK
ZA01-D10A	VDNKFNKEFAHARWWEIRKLPNLNQYQKEAFKTSLKDDPSQSA NLLAEAKKLNDAQAPK
ZA01-H11A	VDNKFNKEFDAARWWEIRKLPNLNQYQKEAFKTSLKDDPSQSA NLLAEAKKLNDAQAPK

ZA01-R13A	VDNKFNKEFDHAAWWEIRKLPNLNQYQKEAFKTSLKDDPSQSA NLLAEAKKLNDAQAPK
ZA01-W14A	VDNKFNKEFDHARAWEIRKLPNLNQYQKEAFKTSLKDDPSQSA NLLAEAKKLNDAQAPK
ZA01-R18A	VDNKFNKEFDHARWWEIAKLPNLNQYQKEAFKTSLKDDPSQSA NLLAEAKKLNDAQAPK
ZA01-K19A	VDNKFNKEFDHARWWEIRALPNLNQYQKEAFKTSLKDDPSQSA NLLAEAKKLNDAQAPK
ZA01-Q25A	VDNKFNKEFDHARWWEIRKLPNLNAYQKEAFKTSLKDDPSQSA NLLAEAKKLNDAQAPK
ZA01-K28A	VDNKFNKEFDHARWWEIRKLPNLNQYQAEAFKTSLKDDPSQSA NLLAEAKKLNDAQAPK
ZA01-E29A	VDNKFNKEFDHARWWEIRKLPNLNQYQKAAFKTSLKDDPSQSA NLLAEAKKLNDAQAPK
ZA01-K32A	VDNKFNKEFDHARWWEIRKLPNLNQYQKEAFATSLKDDPSQSA NLLAEAKKLNDAQAPK
ZA01-T33A	VDNKFNKEFDHARWWEIRKLPNLNQYQKEAFKASLKDDPSQSA NLLAEAKKLNDAQAPK
ZA01-K36A	VDNKFNKEFDHARWWEIRKLPNLNQYQKEAFKTSLADDPSQSA NLLAEAKKLNDAQAPK

Supplementary Table 6: Sequences of the double mutants of Z_{A01} evaluated.

Name	Sequence
ZA01-13A15E	VDNKFNKEFDHAAWEEIRKLPNLNQYQKEAFKTSLKDDPSQSAN LLAEAKKLNDAQAPK
ZA01-13A15N	VDNKFNKEFDHAAWNEIRKLPNLNQYQKEAFKTSLKDDPSQSAN LLAEAKKLNDAQAPK
ZA01-13A18A	VDNKFNKEFDHAAWWEIAKLPNLNQYQKEAFKTSLKDDPSQSAN LLAEAKKLNDAQAPK
ZA01-13A19A	VDNKFNKEFDHAAWWEIRALPNLNQYQKEAFKTSLKDDPSQSAN LLAEAKKLNDAQAPK
ZA01-13A25A	VDNKFNKEFDHAAWWEIRKLPNLNAYQKEAFKTSLKDDPSQSAN LLAEAKKLNDAQAPK
ZA01-15E18A	VDNKFNKEFDHARWEEIAKLPNLNQYQKEAFKTSLKDDPSQSAN LLAEAKKLNDAQAPK
ZA01-15E19A	VDNKFNKEFDHARWEEIRALPNLNQYQKEAFKTSLKDDPSQSAN LLAEAKKLNDAQAPK
ZA01-15E25A	VDNKFNKEFDHARWEEIRKLPNLNAYQKEAFKTSLKDDPSQSAN LLAEAKKLNDAQAPK

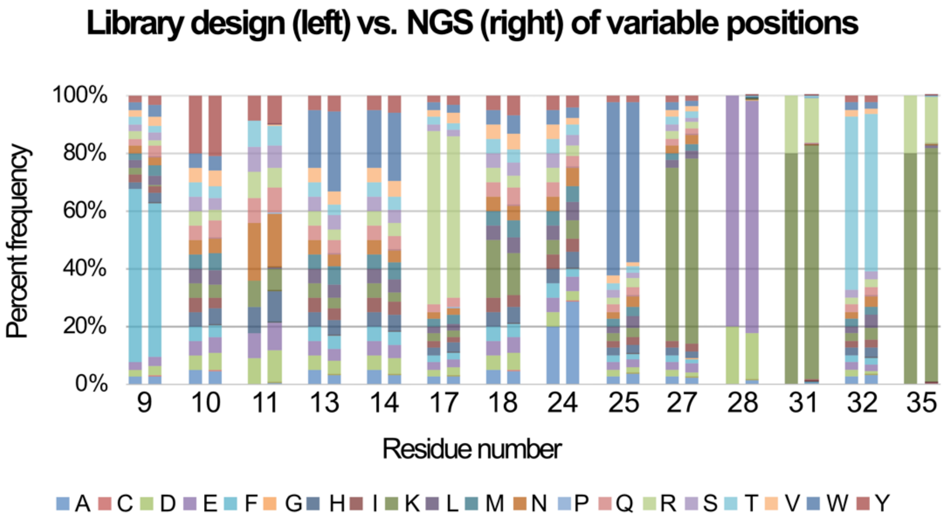

Supplementary Figure 1: Comparison between the designed (left) and measured (right) amino acid distributions across the variable amino acid positions in our maturation library after MiSeq sequencing.

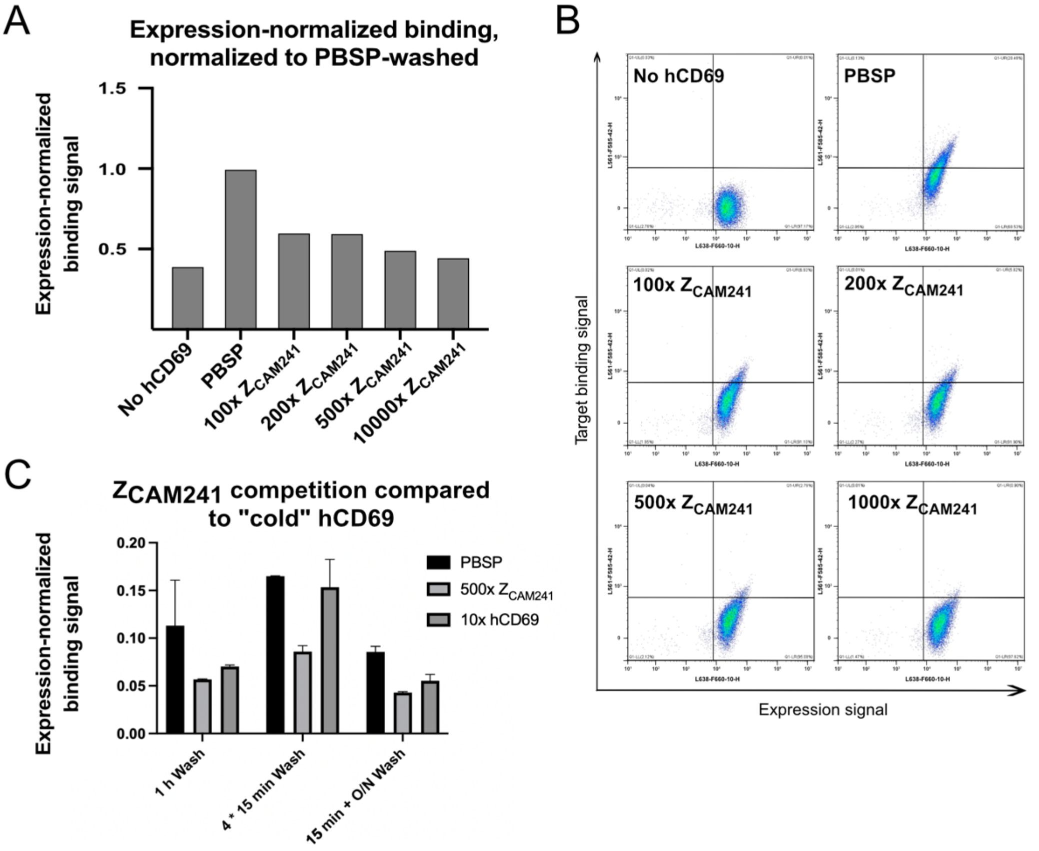

Supplementary Figure 2: Preliminary *E. coli* display experiments on a ZCAM241-displaying clone demonstrating the feasibility of applying selective pressure on hCD69 binding by incubating affibody-displaying *E. coli* with excess amounts of ZCAM241 post hCD69 incubation. **(A)** The expression-normalized hCD69 binding of a ZCAM241-displaying clone as a function of the molar ZCAM241 excess added during off-rate incubation for 1 h at 37°C. Up to and including target incubation with 25 nM hCD69, regular *E. coli* display as described in materials & methods had been performed for all samples (1 h induction, 0.1% L-arabinose). The x-axis labels denote what molar excess of Z_{CAM241} was added during off-rate incubation. **(B)** Dot plots from the same experiment as in (A). X-axis corresponds to display level, y-axis corresponds to target binding level according to the logic laid out in Figure 1B. **(C)** Comparison of the ZCAM241 washing strategy with an economically feasible excess of unbiotinylated hCD69 instead, using three different washing times. Up to and including target incubation with 10 nM hCD69-bio, regular *E. coli* display according to 1.1 had been performed for all samples (1 h induction, 0.1% L-arabinose). Duplicate flow cytometry samples from one experiment were recorded.

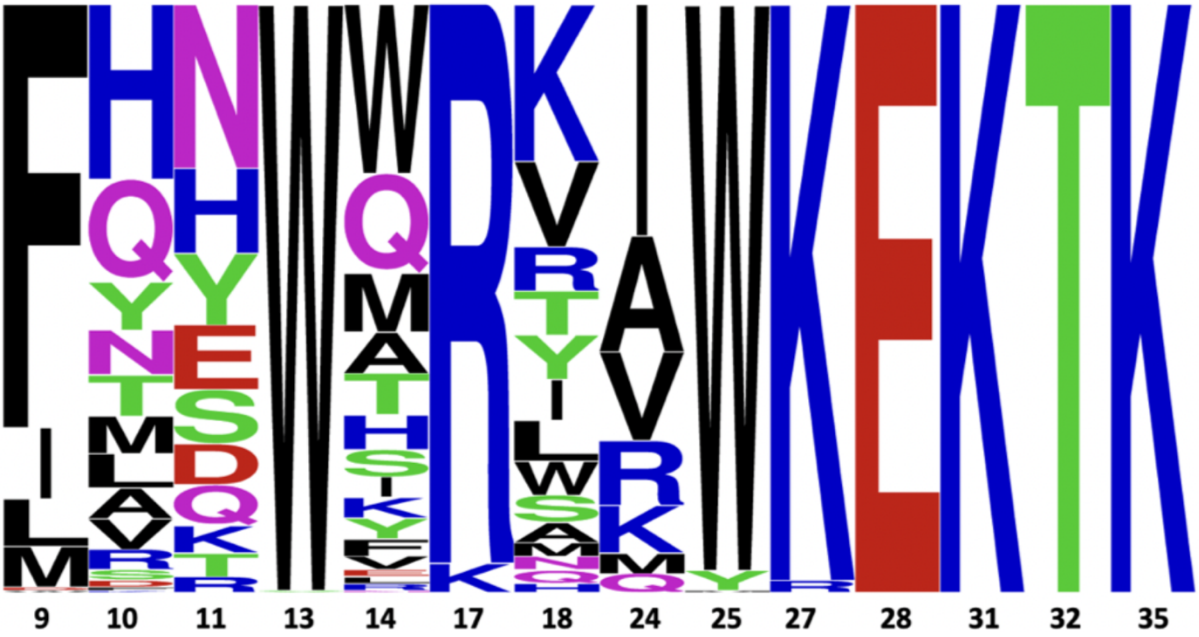

Supplementary Figure 3: The variability of the FACS round 3 output across variable affibody positions. Letter heights are proportional to the frequency of each amino acid in each position. Sequence logo generated using WebLogo (1).

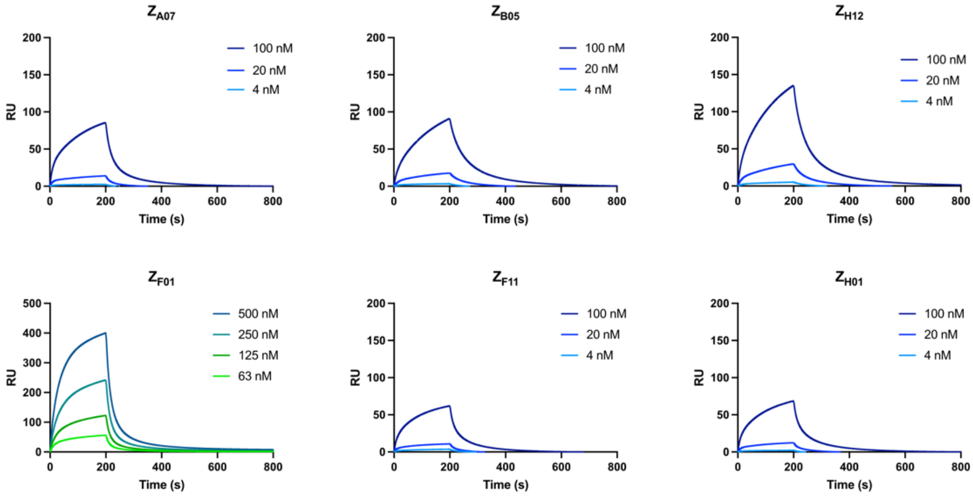

Supplementary Figure 4: SPR sensorgrams from the candidate affinity screen after FACS 4, injecting them in Z-H_6 format over immobilized hCD69. Remaining candidates are displayed in Figure 3A.

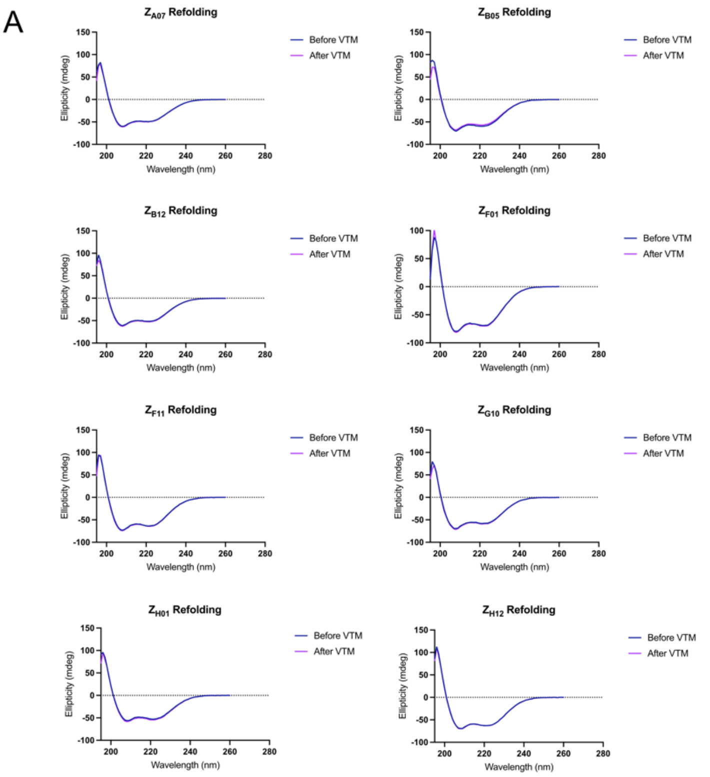

B

Construct	T_m (°C)
Z_{A01}	44
Z_{A07}	55
Z_{B05}	48
Z_{B12}	55
Z_{F01}	57
Z_{F11}	54
Z_{G10}	48
Z_{H01}	49
Z_{H12}	58

Supplementary Figure 5: CD spectroscopy data for single clones in the FACS 4 output. **(A)** Overlapped CD spectra before and after thermal denaturation for the candidates from FACS 4, indicating complete refolding for all affibody variants. Z_{A01}:s spectra are shown in Figure 3B. **(B)** Melting temperatures of FACS 4 output clones as determined by variable temperature measurements (VTM:s). VTM curves are shown in Figure 3C.

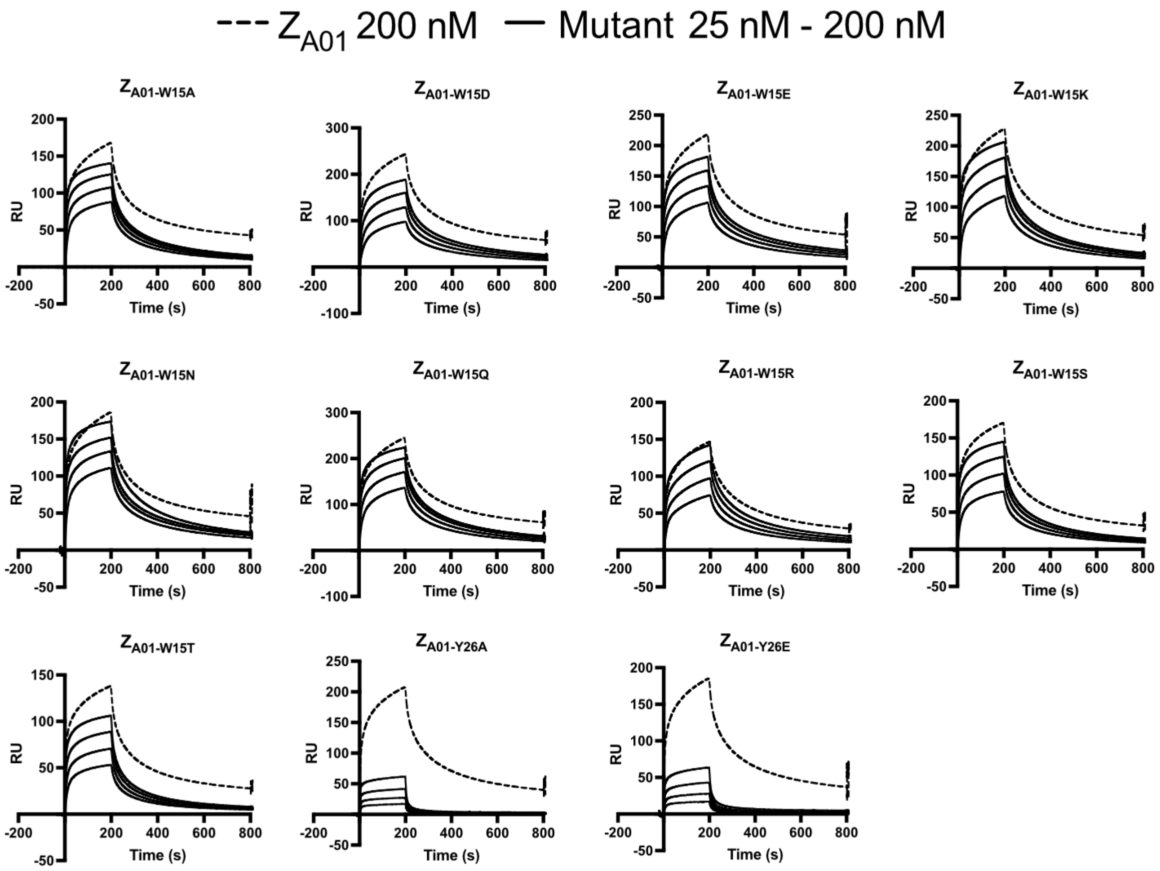

Supplementary Figure 6: SPR sensorgrams from the protein language algorithm-based Z_{A01} single mutants (full lines), injected in Z-H_6 format over immobilized hCD69, compared with Z_{A01} (dashed lines) injected over the same Biacore 8K flow cell.

A

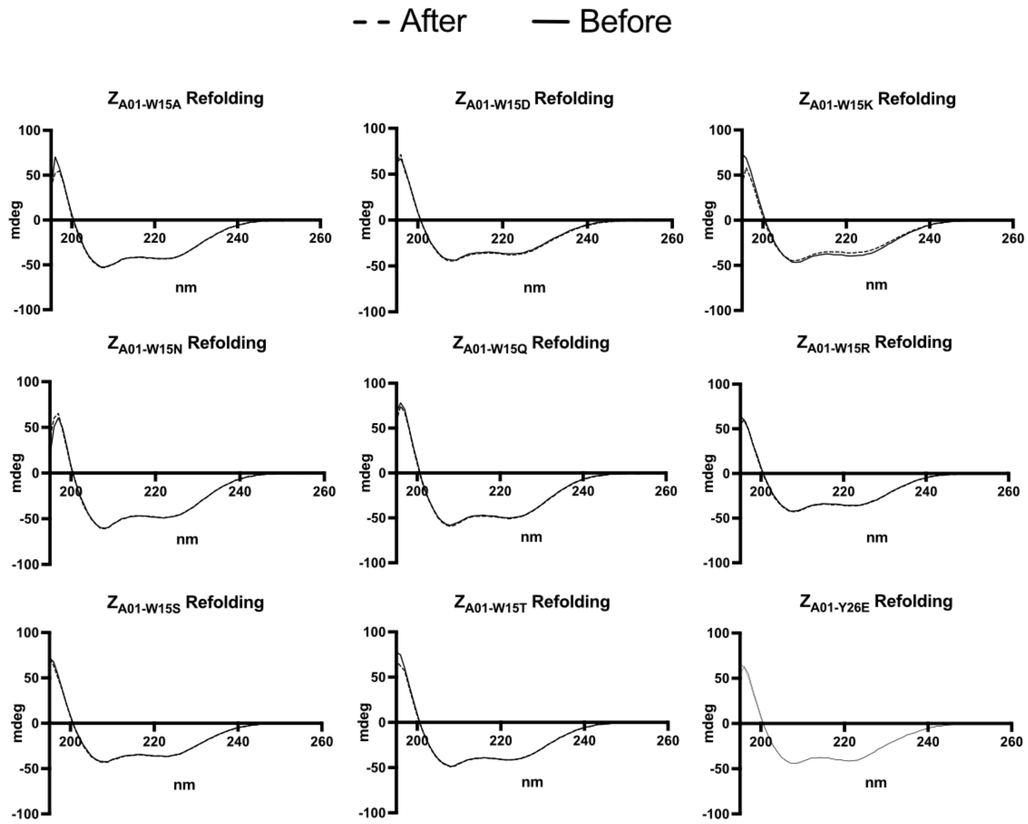

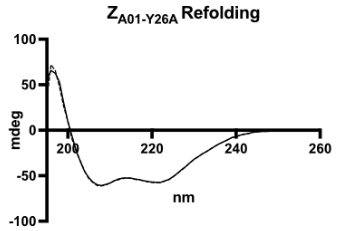

B

Construct	T_m (°C)	Construct	T_m (°C)
$Z_{A01\text{-}W15A}$	48	$Z_{A01\text{-}W15R}$	44
$Z_{A01\text{-}W15D}$	51	$Z_{A01\text{-}W15S}$	46
$Z_{A01\text{-}W15E}$	53	$Z_{A01\text{-}W15D}$	45
$Z_{A01\text{-}W15K}$	43	$Z_{A01\text{-}Y26A}$	47
$Z_{A01\text{-}W15N}$	49	$Z_{A01\text{-}Y26E}$	47
$Z_{A01\text{-}W15Q}$	47		

Supplementary Figure 7: CD spectroscopy data for Z_{A01} single mutants based on machine learning. **(A)** Overlapped CD spectra before and after thermal denaturation for the candidates from Z_{A01} single mutants based on the protein language model, showing complete refolding for all mutants. $Z_{A01\text{-}W15E}$:s spectra are shown in Figure 4B. **(B)** T_m:s of protein language model-guided mutants as determined by VTM:s. VTM curves are shown in Supplementary Figure 13A.

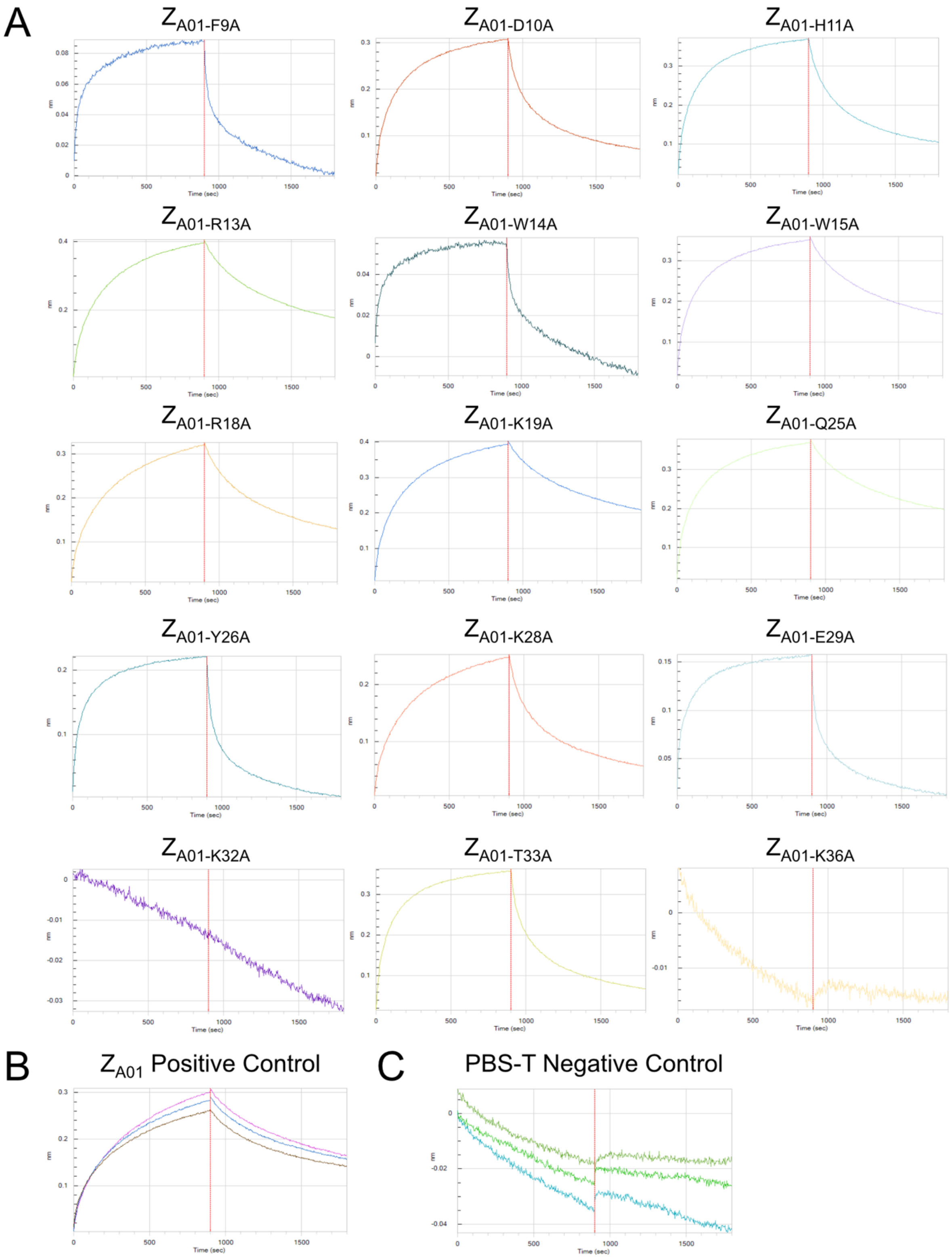

Supplementary Figure 8: BLI data from the alanine scan of Z_{A01}. **(A)** Sensorgrams for the 15 alanine mutants incubating hCD69-conjugated sensor tips in 500 nM of the respective binders. **(B)** In each analysis (the 15 binders were evaluated in sets of five), one hCD69-conjugated sensor tip was incubated with Z_{A01} as a positive control. **(C)** In each analysis, one hCD69-conjugated sensor tip was incubated with pure PBS-T as a negative control.

A

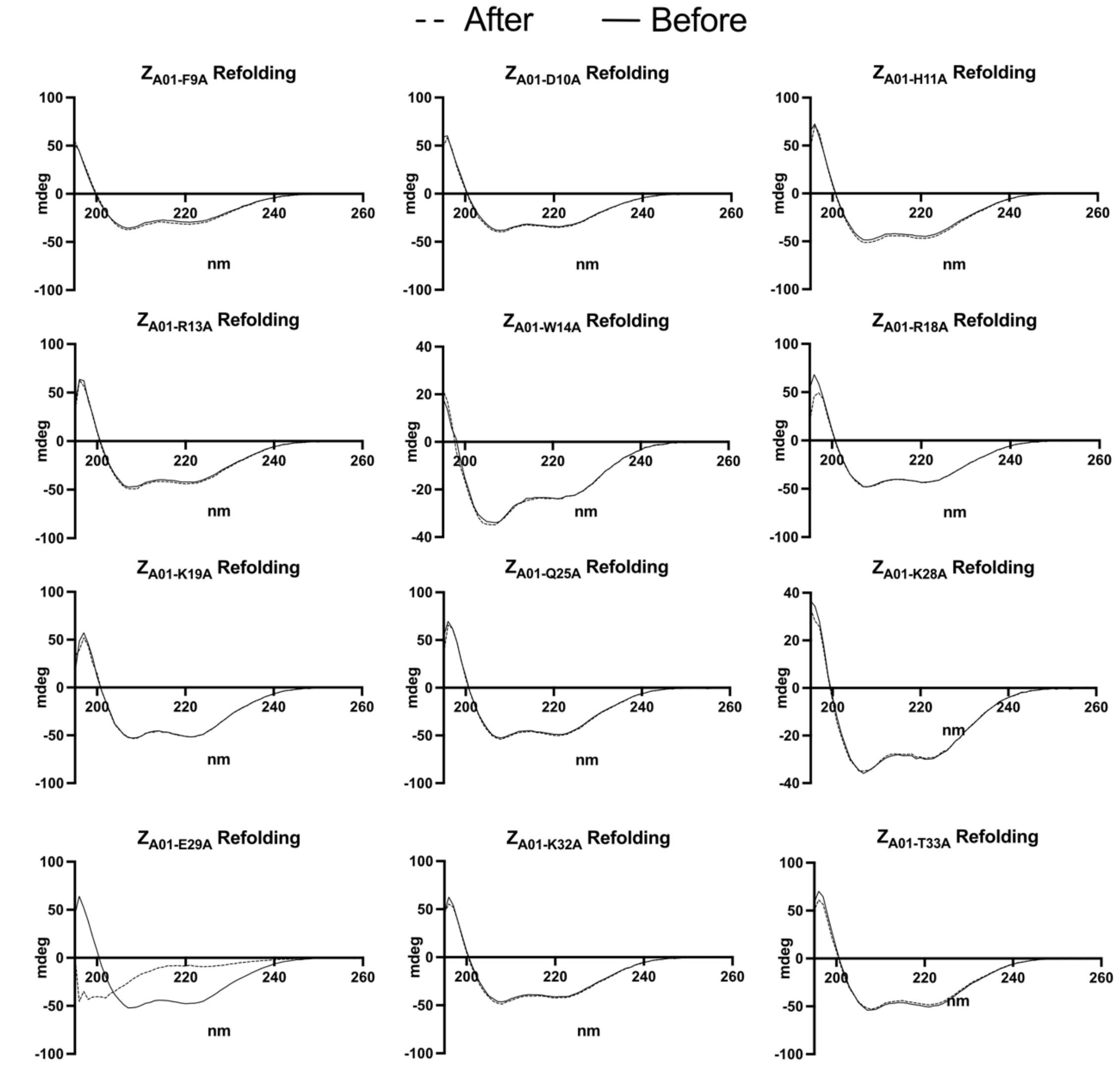

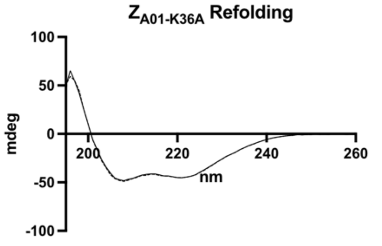

B

Construct	T_m (°C)	Construct	T_m (°C)
$Z_{A01\text{-}F9A}$	35	$Z_{A01\text{-}Q25A}$	45
$Z_{A01\text{-}D10A}$	37	$Z_{A01\text{-}K28A}$	31
$Z_{A01\text{-}H11A}$	41	$Z_{A01\text{-}E29A}$	38
$Z_{A01\text{-}R13A}$	57	$Z_{A01\text{-}K32A}$	41
$Z_{A01\text{-}W14A}$	28	$Z_{A01\text{-}T33A}$	46
$Z_{A01\text{-}R18A}$	50	$Z_{A01\text{-}K36A}$	45
$Z_{A01\text{-}K19A}$	46		

Supplementary Figure 9: CD spectroscopy data for Z_{A01} alanine scan variants. **(A)** Overlapped CD spectra before and after thermal denaturation for the candidates from the Z_{A01} alanine scan, showing complete refolding except for $Z_{A01\text{-}E29A}$. **(B)** T_m:s of alanine scan variants as determined by VTM:s. VTM curves are shown in Supplementary Figure 13B.

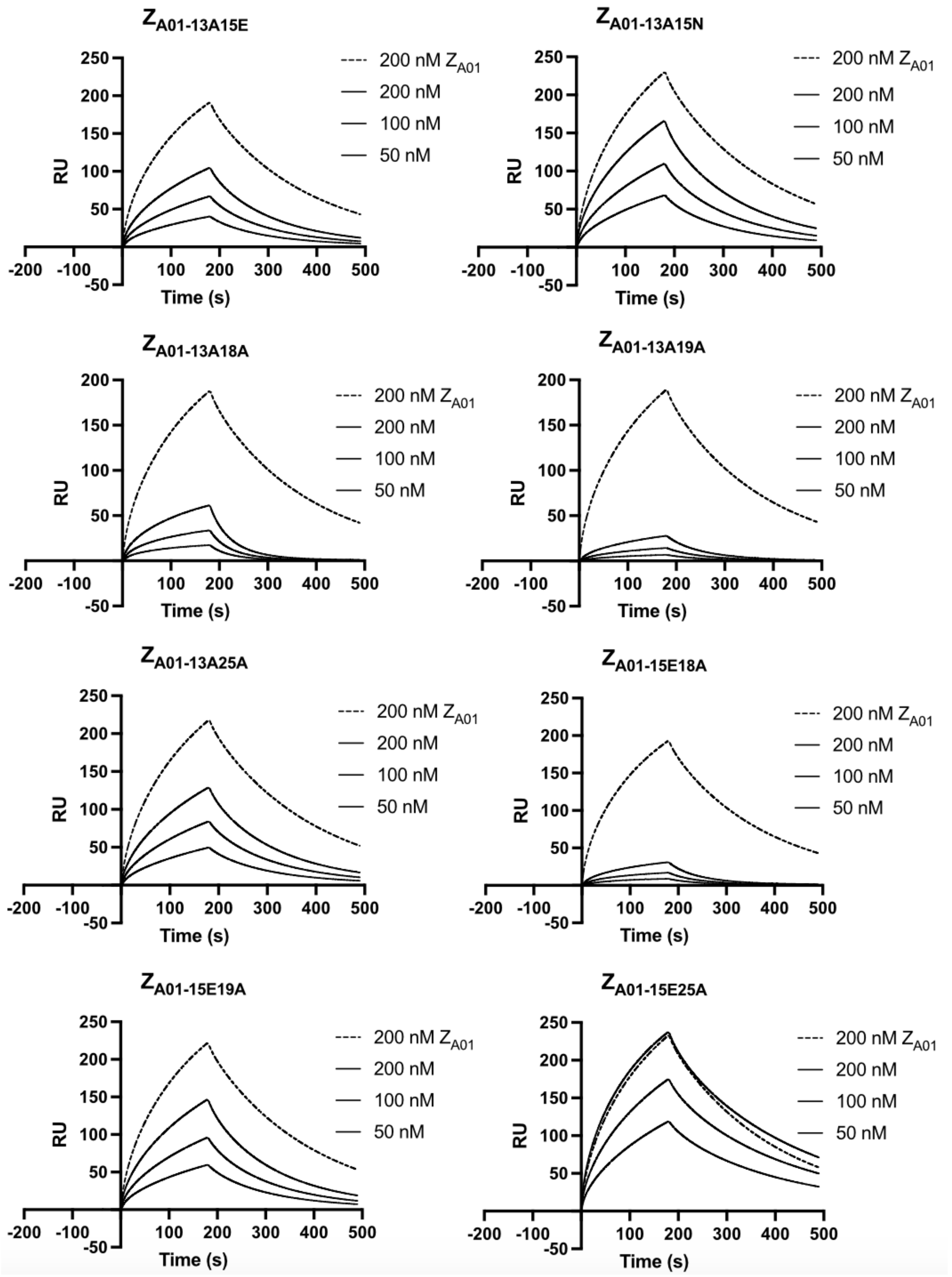

Supplementary Figure 10: SPR sensorgrams from a Biacore 8K experiment of the Z_{A01} double mutants (full lines), inject them in Z-H_6 format over immobilized hCD69, compared with Z_{A01} (dashed lines) injected over the same flow cell.

A

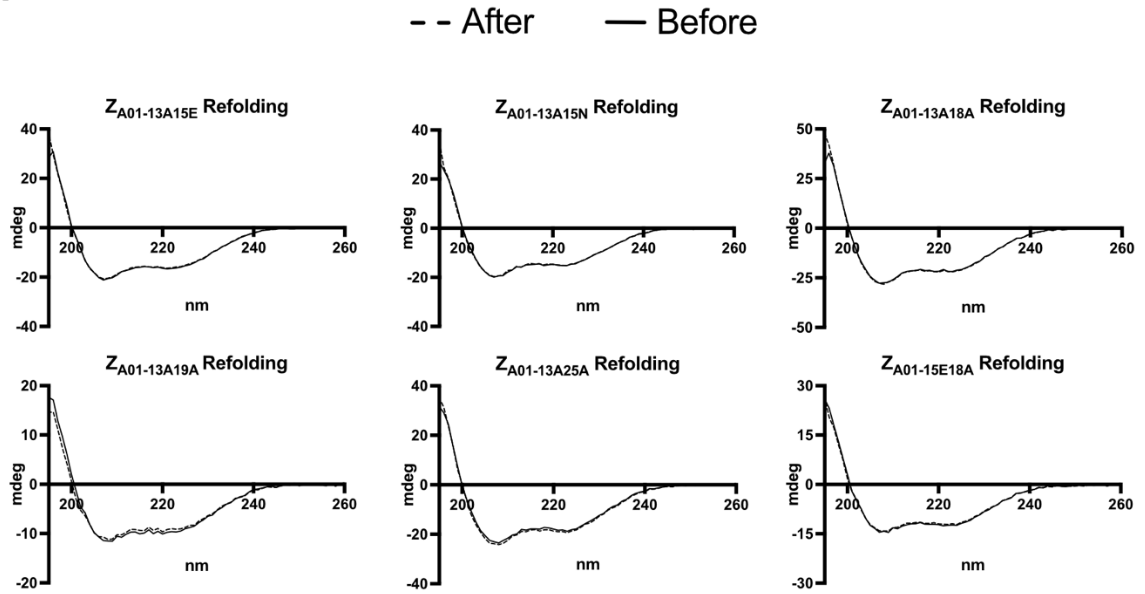

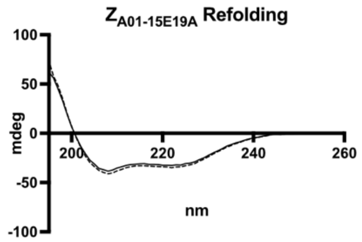

B

Construct	T_m (°C)	Construct	T_m (°C)
$Z_{A01-13A15E}$	65	$Z_{A01-13A25A}$	65
$Z_{A01-13A15N}$	60	$Z_{A01-15E18A}$	53
$Z_{A01-13A18A}$	65	$Z_{A01-15E19A}$	52
$Z_{A01-13A19A}$	63		

Supplementary Figure 11: CD spectroscopy data for Z_{A01} double mutants. **(A)** Overlapped CD spectra before and after thermal denaturation demonstrating the desired refolding behavior for all samples. **(B)** T_m:s of double mutants as determined by VTM:s. VTM curves are shown in Supplementary Figure 13C.

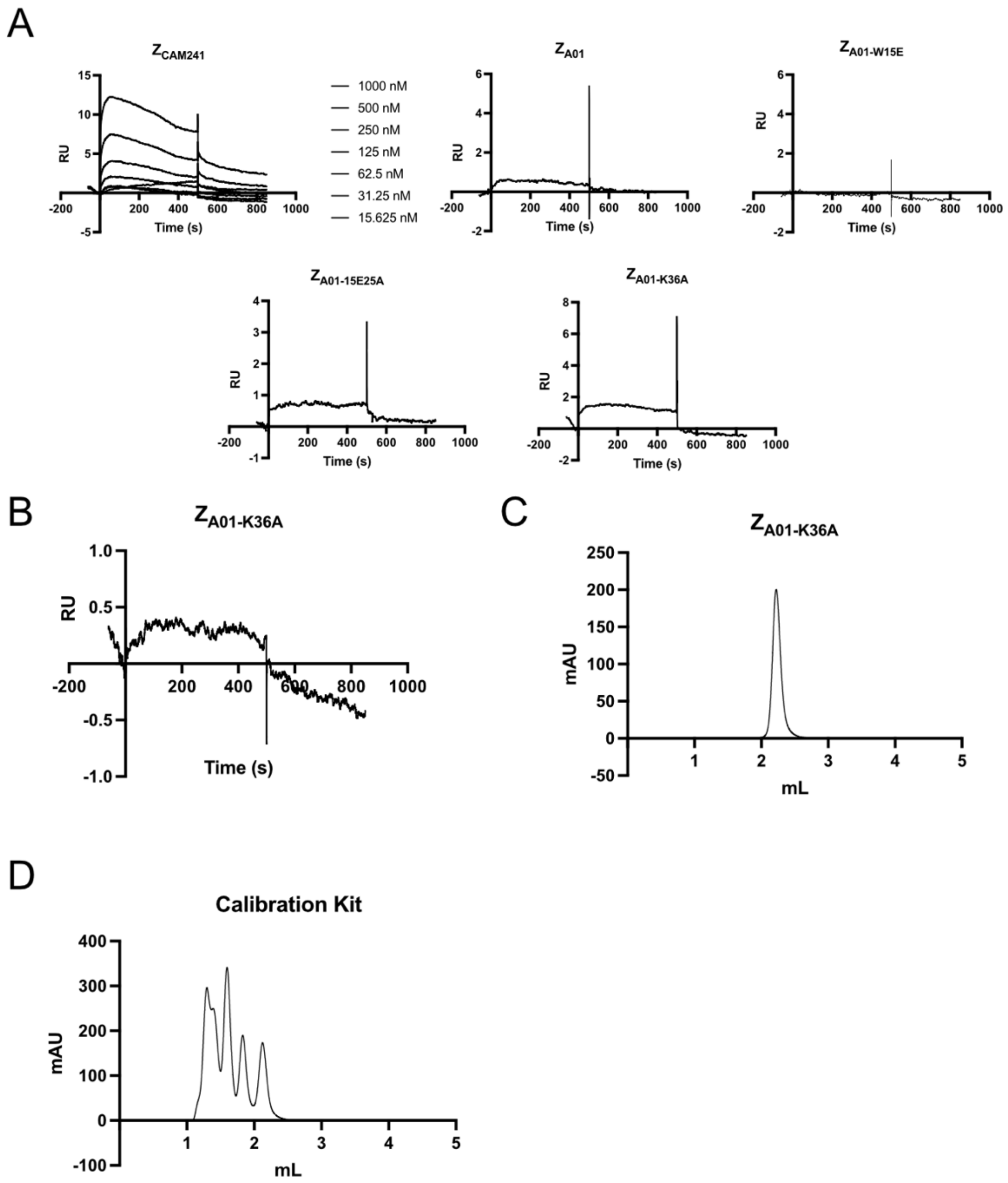

Supplementary Figure 12: (A) SPR controls for Fc interaction of binders analyzed on Biacore T200 for binding to hCD69-Fc. Here, binders were injected over a control surface with immobilized CLEC4C-Fc. Only the highest concentrations analyzed towards hCD69-Fc are shown (1 µM for $Z_{A01\text{-}K36A}$ and 64 nM for the remainder) except for ZCAM241. **(B)** SPR verification of $Z_{A01\text{-}K36A}$ as negative control, here injected over hCD69-Fc at 1 µM concentration. **(C)** SEC chromatogram for $Z_{A01\text{-}K36A}$. **(D)** SEC chromatogram for the calibration kit employed, peaks correspond to molecular weights 75, 44, 29, 13.7 and 6.5 kDa, from left to right.

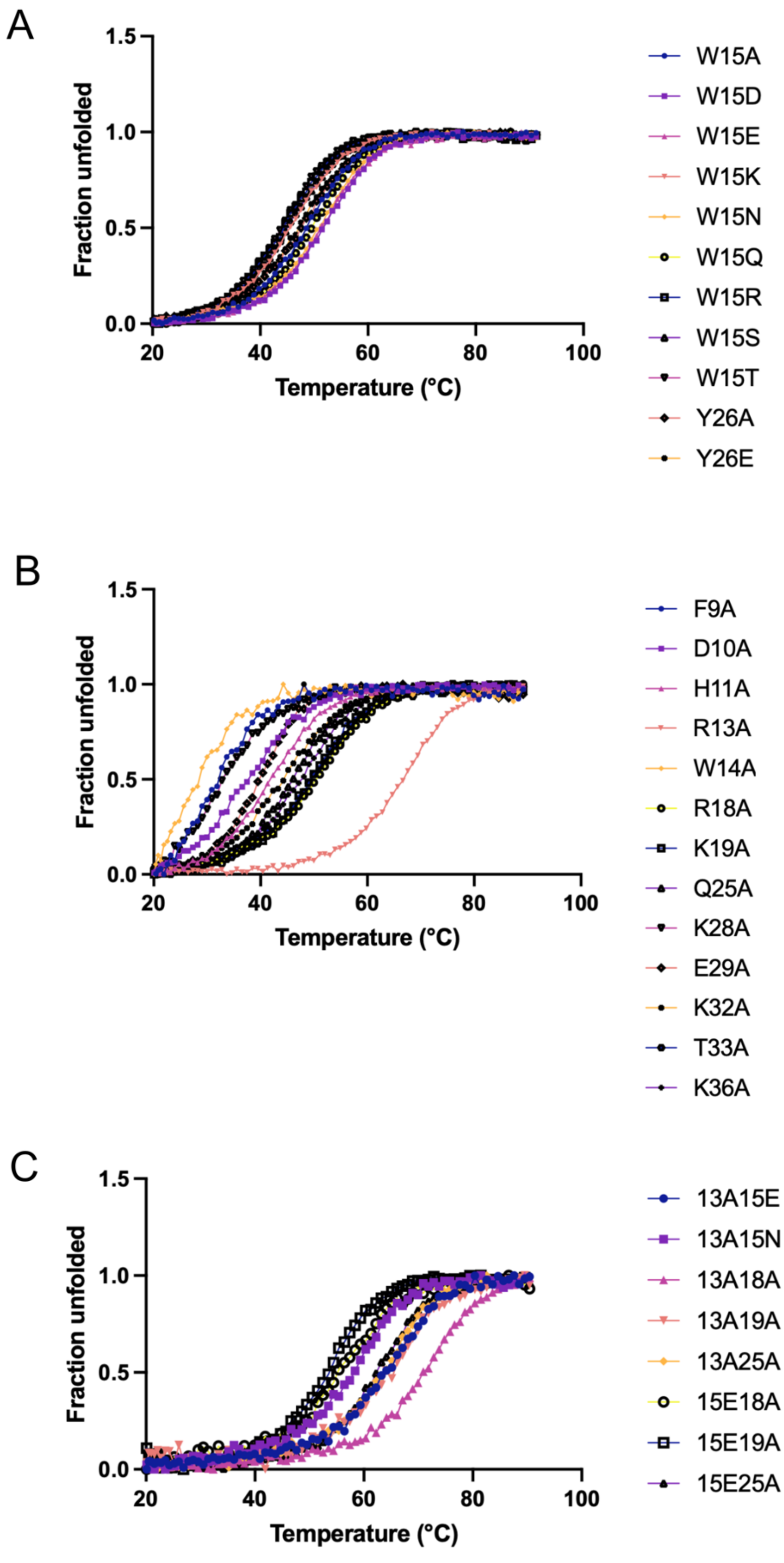

Supplementary Figure 13: VTM curves for Z_{A01} mutants. **(A)** Protein language model-based single mutants. **(B)** Alanine scan mutants. **(C)** Double mutants.

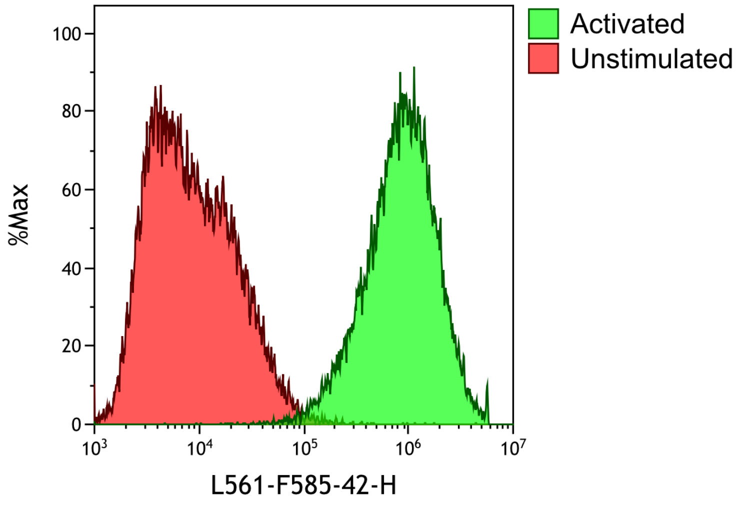

Supplementary Figure 14: Overlay histogram showing representative samples of unstimulated and activated Jurkat cells stained with a fluorophore-coupled anti-CD69 IgG.

Supplementary References

1. Crooks GE, Hon G, Chandonia JM, Brenner SE. WebLogo: A sequence logo generator. Genome Res. 2004 Jun;14(6):1188–90.
